# Gut microbiota‐mediated immune and metabolic dysregulation in coronary artery disease progression and prognosis

**DOI:** 10.1002/imt2.70147

**Published:** 2026-07-01

**Authors:** Honghong Liu, Siqin Feng, Lifeng Liang, Muyun Tang, Yifei Wang, Yiyang Wang, Zhiyu Zhang, Haiping Xing, Chenhong Zhang, Ran Tian, Shuyang Zhang

**Affiliations:** ^1^ Department of Internal Medicine, Peking Union Medical College Hospital Chinese Academy of Medical Sciences & Peking Union Medical College Beijing China; ^2^ Department of Cardiology, Peking Union Medical College Hospital Chinese Academy of Medical Sciences & Peking Union Medical College Beijing China; ^3^ State Key Laboratory of Complex Severe and Rare Diseases, Peking Union Medical College Hospital Chinese Academy of Medical Sciences & Peking Union Medical College Beijing China; ^4^ 01Life Institute Shenzhen China; ^5^ State Key Laboratory of Microbial Metabolism, School of Life Sciences and Biotechnology Shanghai Jiao Tong University Shanghai China; ^6^ Joint International Research Laboratory of Metabolic & Developmental Science Shanghai Jiao Tong University Shanghai China

## Abstract

The gut microbiota has emerged as a metabolically active endocrine‐like organ with a crucial role in coronary artery disease (CAD), yet its contribution to adverse clinical outcomes remains incompletely understood. In this prospective cohort of 319 participants, we integrated metagenomic and metabolomic profiling with longitudinal follow‐up over a median of 1.85 years. Fecal microbiota transplantation from patients with CAD transmitted susceptibility to atherosclerosis in antibiotic‐treated *ApoE* ^−/−^ mice, accompanied by microbiota‐induced vascular inflammation mediated through LPS–TLR4 signaling. Gut microbiota‐derived aromatic amino acid metabolism was associated with thrombotic risk and major adverse cardiac events (MACE). Machine learning identified gut microbial features that improved prospective prediction of MACE. Together, these findings implicate the gut microbiome in CAD progression and support the development of microbiome‐based strategies for cardiovascular risk prediction and prevention.

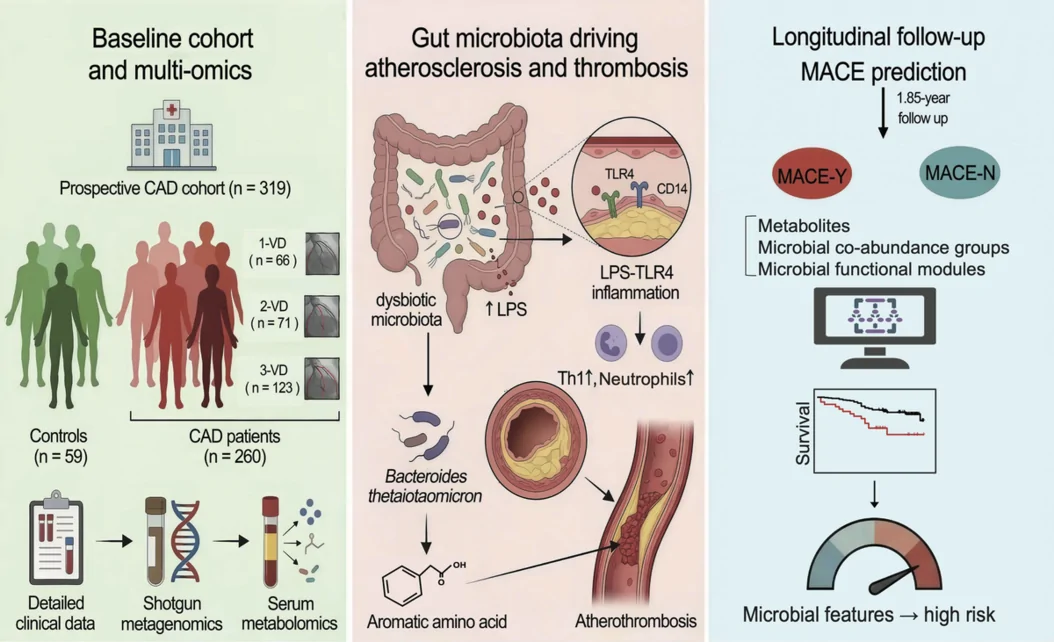

## AUTHOR CONTRIBUTIONS

**Honghong Liu**: Conceptualization; methodology; investigation; funding acquisition; writing—original draft; visualization; project administration. **Siqin Feng**: Methodology; data curation. **Lifeng Liang**: Software; formal analysis. **Muyun Tang**: Data curation; methodology. **Yifei Wang**: Investigation; methodology. **Yiyang Wang**: Investigation; methodology. **Zhiyu Zhang**: Validation; data curation. **Haiping Xing**: Validation; data curation. **Chenhong Zhang**: Formal analysis; project administration; writing—review and editing; supervision. **Ran Tian**: Project administration; conceptualization. **Shuyang Zhang**: Resources; supervision; project administration; funding acquisition; writing—review and editing.

## CONFLICT OF INTEREST STATEMENT

The authors declare no conflicts of interest.

## ETHICS STATEMENT

This metagenomics CAD cohort was a single‐center, prospective study approved by the Peking Union Medical College Hospital Board. The study was approved by local ethics committees (JS‐1195, Peking Union Medical College Hospital, Beijing), and informed consent was obtained from all subjects. All mice in our study were generated and provided by the Institute of Laboratory Animal Sciences (ILAS) at the Chinese Academy of Medical Sciences and Peking Union Medical College [research license no. SYXK (Beijing) 2015‐0035], which is a member of (and accredited by) the American Association for the Accreditation of Laboratory Animal Care. All animal protocols were approved by the Ethics Committee for Animal Care and Use at the Institute Laboratory Animal Science (IACUC No. NHT17002).

To the Editor,

Coronary artery disease (CAD) remains a leading contributor to the global burden of disease, accounting for approximately 9.1 million deaths annually. CAD is a chronic and dynamic pathological process in which clinically stable coronary plaque may progress to acute myocardial infarction (MI) or sudden cardiac death through occlusive thrombosis [1]. Despite advances in cardiovascular prevention and treatment, early identification of individuals at heightened risk of adverse thrombotic events remains challenging, largely because sensitive biomarkers and predictive strategies for early‐stage disease are still limited.

Accumulating evidence implicates the gut microbiome as a non‐traditional contributor to CAD pathogenesis [2, 3]. Microbial influences on cardiovascular disease are thought to be mediated through both direct metabolic pathways and indirect mechanisms involving microorganism‐associated molecular patterns [4]. Microbial‐dependent metabolites, including trimethylamine N‐oxide, short‐chain fatty acids, and aromatic amino acids, have been extensively investigated in cardiovascular disease, with emerging roles in vascular homeostasis, immune regulation, and platelet aggregation [5, 6, 7]. In parallel, gut microbiota‐derived lipopolysaccharide (LPS) may promote metabolic endotoxemia, thereby contributing to vascular inflammation, atherogenesis, and ischemic events [8].

Although several cross‐sectional studies have characterized gut microbiota alterations in patients with CAD [2, 9], longitudinal evidence linking the gut microbiome to cardiovascular prognosis remains limited. Building on our previously established PUMCH‐CAD cohort, a registered prospective cohort study (ChiCTR2000033897, http://www.chictr.org.cn), we integrated metagenomic, metabolomic, and clinical approaches to evaluate the prognostic relevance of CAD‐associated microbial features. We further investigated whether disease‐associated microbiota may contribute causally to atherosclerosis progression using fecal microbiota transplantation. Together, these analyses aimed to define a microbiome‐based signature of CAD severity and prognosis, and to establish a translational framework for incorporating metagenomic profiling into cardiovascular risk stratification.

## CLINICAL CHARACTERISTICS OF THE CAD COHORT

We enrolled 59 control individuals and 260 patients with CAD according to predefined selection criteria described in the Methods. To quantify atherosclerotic burden and disease severity, all patients underwent coronary angiography and were stratified by at least two blinded cardiologists according to the number of stenosed vessels, defined as >50% luminal stenosis: one‐vessel disease (1‐VD, *n* = 66), two‐vessel disease (2‐VD, *n* = 71), or three‐vessel disease (3‐VD, *n* = 123). Consistent with this stratification, both the Gensini score [10] and SYNTAX score [11] increased progressively with the number of stenosed vessels, indicating a graded increase in atherosclerotic burden (Figure S1). The proportion of patients with myocardial infarction also increased across CAD subgroups (1‐VD 12.1%; 2‐VD 28.2%; 3‐VD 26.0%), accompanied by higher circulating levels of cardiac troponin (cTnI), a conventional biomarker of myocardial injury (1‐VD 0.004, 0–0.0223 μg/L; 3‐VD 0.009, 0–0.072 μg/L; *p* = 0.037). In total, 39 clinical variables were collected, including demographics, comorbidities, medication history, and laboratory parameters (Table S1). Compared with patients with CAD, control individuals had lower levels of established cardiovascular risk factors, including age, smoking exposure, blood pressure, and body mass index. Information on gastrointestinal status, physical activity, and dietary patterns was also collected to account for potential microbiome‐related confounders (Table S2).

### MICROBIAL VARIATION SHIFTS WITH CAD SEVERITY AS REFLECTED IN CO‐ABUNDANCE GROUPS

**Figure 1:**
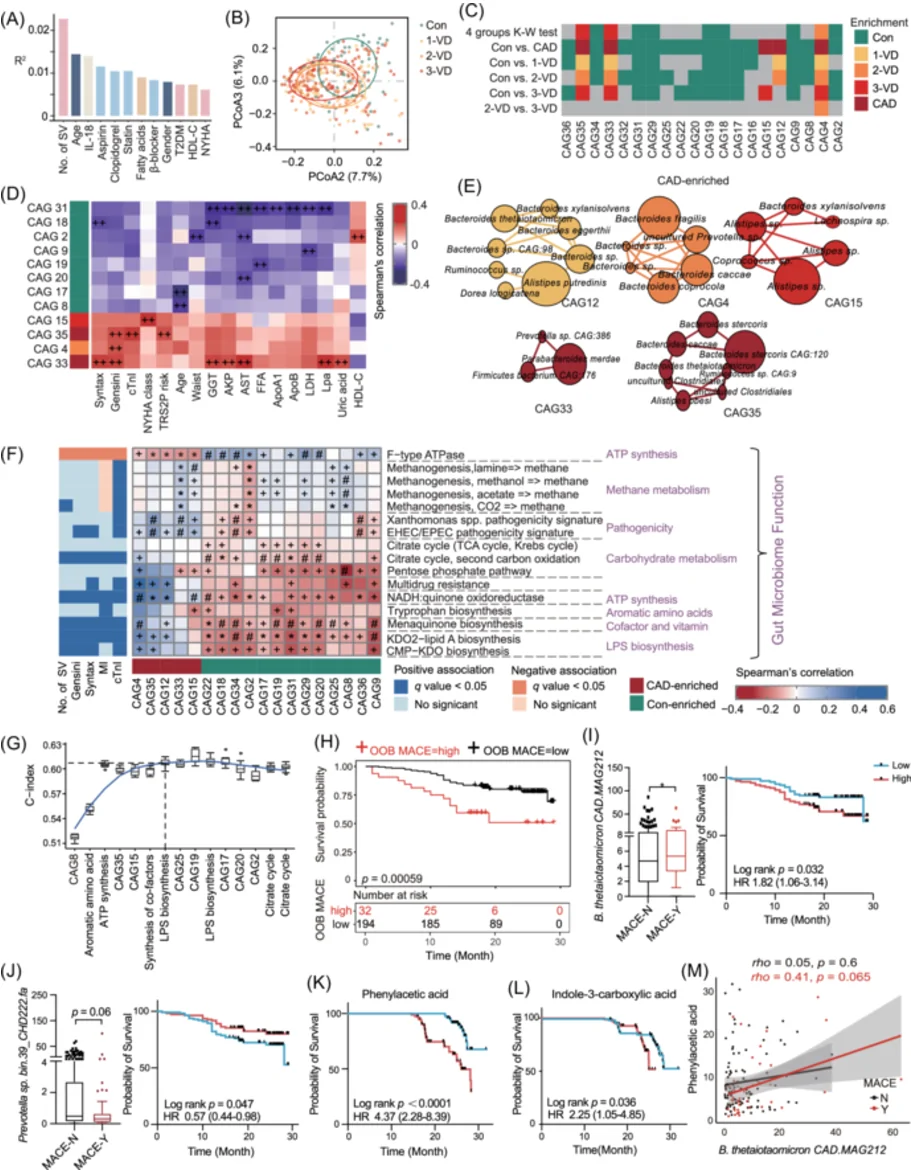
Gut microbiota features associated with coronary artery disease severity and prognostic microbial signatures for major adverse cardiac events (MACE). (A) Bar plot illustrating the host factors significantly associated with variations in gut microbiome composition, calculated from between‐sample Bray–Curtis distances. Effect sizes and statistical significance were assessed using PerManova/Adonis, with significance defined as *p* < 0.05. Bars were colored according to metadata categories. No. of SV, Number of stenosed coronary vessels; IL‐18, interleukin‐18; T2DM, type 2 diabetes mellitus; HDL‐C, High‐density lipoprotein cholesterol; NYHA, New York Heart Association class for cardiac function. (B) Principal coordinates analysis (PCoA) based on MAG‐level Bray–Curtis dissimilarity. Differences in beta‐diversity were tested using PerManova with 9999 permutations. Con, Control; Patients were stratified by the number of stenosed coronary arteries on angiography into single‐vessel disease (1‐VD), double‐vessel disease (2‐VD), and triple‐vessel disease (3‐VD). (C) Co‐abundance groups (CAGs) that differed significantly across four study groups or between CAD subgroups. *p* values were adjusted across the tested CAGs within each comparison using the Benjamini–Hochberg false discovery rate (FDR) method. Only CAGs with FDR‐adjusted *q* < 0.05 in at least one indicated comparison are shown. (D) Spearman correlation analysis between CAGs and major CAD‐related clinical indicators. Color indicates the direction and magnitude of the correlation, with red denoting positive correlations and blue denoting negative correlations. Statistical significance after FDR corrections is indicated as follows: ++ *q* < 0.05. GGT, gamma‐glutamyl transferase; AKP, alkaline phosphatase; AST, aspartate aminotransferase; FFA, free fatty acids; ApoA1, apolipoprotein A1; ApoB, apolipoprotein B; LDH, lactate dehydrogenase. (E) MAG‐level co‐abundance network showing MAG enrichment across different stages within significantly altered CAGs. Node size indicates the mean relative abundance of each MAG. Taxonomic labels indicate the lowest confidently assigned taxonomic level. Lines between nodes represent correlations between MAGs. Only correlations with an absolute coefficient >0.4 are shown. (F) Fine‐grained correlation profile integrating microbial functional modules, CAGs, and clinical traits across 319 individuals. The left panel shows associations between microbial functional modules and clinical phenotypes, with color indicating the direction of association. The middle panel shows associations between the same modules and CAGs, with color representing Spearman correlation coefficients. Statistical significance after FDR correction is indicated as follows: + *q* < 0.1, # *q* < 0.01, * *q* < 0.001. (G) Construction and internal evaluation of the random survival forest (RSF) model using ensemble permutation importance—guided forward selection of candidate microbial prognostic features. C‐index values were computed using out‐of‐bag (OOB)‐predicted MACE risk scores from 1000 iterations of RSF modeling. The blue line indicates local regression fitting, and the dotted line marks the selected predictor set with the highest median OOB C‐index. (H) Kaplan–Meier curves of MACE risk groups dichotomized according to OOB‐predicted microbial risk scores derived from the final RSF model. (I) and (J) Relative abundance of *Bacteroides thetaiotaomicron* CAD. MAG212 or *Prevotella sp*. in CAD patients with or without MACE. MACE‐Y and MACE‐N indicate patients with and without major adverse cardiac events, respectively. Boxes represent the inter‐quartile ranges, and lines inside the boxes denote medians. Kaplan–Meier curves show event‐free survival according to high versus low abundance quartiles of the corresponding bacterial taxa. Kaplan–Meier estimates of MACE‐free survival according to high versus low serum level quartiles of phenylacetic acid (K) and indole‐3‐carboxylic acid (L). (M) Spearman correlation analysis between serum phenylacetic acid level and the abundance of *B. thetaiotaomicron* CAD.MAG212 in CAD patients with or without MACE.

Shotgun metagenomic sequencing generated an average of 8.2 Gb of high‐quality reads per sample, yielding a total of 49.054 × 10^9^ bp assembled into 5,858,411 contigs (Table S4). These contigs were further clustered into 1046 metagenome‐assembled genomes (MAGs), representing distinct bacterial populations within the gut microbiome (Figure S2, Table S5). Across the cohort, exploratory univariable PerManova/Adonis screening identified 16 clinical variables, including disease status, demographic characteristics, medication use, inflammatory markers, and lipid parameters, that were associated with variations in gut microbiome composition. Among these variables, the number of stenosed vessels showed a statistically significant but modest association with MAG‐level Bray–Curtis dissimilarity (Figure 1A, Table S6). Consistently, the global structure of the gut microbiota differed markedly between the control individuals and patients across CAD stages, with a significant difference also observed between the 2‐VD and 3‐VD subgroups (*p* = 0.048, PerManova test, Figure 1B).

Because bacterial taxa often function collectively within ecological networks, we constructed a co‐abundance network of MAGs based on SparCC correlation coefficients [12]. The MAGs were subsequently organized into 36 co‐abundant groups (CAGs) across all individuals (Table S7). Fifteen CAGs were enriched in control individuals (Wilcoxon's test, *p* < 0.05), and these groups were predominantly composed of MAGs assigned to *Faecalibacterium* and *Ruminococcus*, accounting for 38.84% and 11.79% of MAGs, respectively. Consistent with observations from diverse healthy cohorts, *Faecalibacterium* spp. showed a dominant pattern in the gut microbiota of healthy adults (Figure S3) [13]. By contrast, CAG4, CAG12, CAG15, CAG33, and CAG35 were significantly enriched in patients with CAD. CAG12 and CAG4, which were mainly composed of *Bacteroides* spp., were elevated from the early stages of CAD, whereas CAG15, characterized by *Alistipes* sp., increased specifically in the 3‐VD subgroup (Figure 1C,E).

Moreover, the CAGs enriched in patients with CAD were positively associated with atherosclerotic burden, cTnI levels, and additional cardiovascular risk indicators, including NYHA class, uric acid, free fatty acids, and TRS 2°P risk score [14] (Figure 1D). Together, these findings indicate that CAD progression is accompanied by coordinated shifts in gut microbial co‐abundance networks, providing a rationale for identifying stage‐specific microbial features as potential targets for prevention, risk stratification, and intervention.

### GUT MICROBIOTA CONTRIBUTE TO CAD THROUGH INFLAMMATORY AND METABOLIC PATHWAYS

Since circulating metabolites often act as intermediaries between the gut microbial ecosystem and host cardiovascular biology, we performed untargeted metabolome profiling on fasting serum samples from a subset of participants (*N* = 152). To identify metabolic features associated with CAD severity, we applied a cross‐comparison strategy across disease strata. This approach yielded an integrated set of 562 metabolite features, which were further clustered into 35 co‐abundance metabotypes (Figure S4A, Table S8). Among these metabotypes, 19 of 35 clusters (54%) showed positive or negative associations with CAD severity indices and cardiovascular risk factors (Figure S4B,C), highlighting broad metabolic remodeling along CAD progression.

To further characterize functional alterations in the CAD‐associated gut microbiome, we constructed a catalog of 1,907,752 non‐redundant microbial genes and annotated them into 279 Kyoto Encyclopedia of Genes and Genomes functional modules, as previously described [15]. Cross‐domain association analysis integrating CAD phenotypes, microbial functional modules, and the CAGs revealed coordinated relationships between microbial ecology and host disease features (Figure 1F, Table S9). Functional modules positively associated with CAD severity included biosynthesis of LPS, cofactors and vitamins, carbohydrate metabolism, and multiple pathogenicity‐related systems. Notably, microbial LPS biosynthesis pathways showed a strong positive correlation with CAD‐enriched CAGs, including CAG4, CAG12, and CAG35. Consistent with these microbial functional changes, serum levels of LPS‐binding protein (LBP) increased progressively with CAD severity (Figure S5), suggesting enhanced exposure to microbial endotoxin along disease progression.

Additionally, microbial tryptophan biosynthesis, a pathway implicated in vascular homeostasis [5], was positively correlated with serum metabotype M06, which comprised benzoate and D‐proline‐related metabolites, and was significantly enriched in MI events (Figure S6). Collectively, these multi‐omics analyses indicate that CAD progression is accompanied by coordinated alterations in microbial inflammatory pathways, particularly LPS biosynthesis and aromatic amino acid metabolism, implicating these microbial functions as potential contributors to disease severity and cardiovascular risk.

### CAD‐SEVERITY‐SPECIFIC MICROBIAL FEATURES PREDICT CARDIOVASCULAR EVENTS DURING FOLLOW‐UP

During a median follow‐up of 1.85 years (interquartile range, 1.5–2.15 years), 43 major adverse cardiac events (MACE, including primary and secondary events with 10 overlaps) occurred among 226 patients with CAD who completed follow‐up, corresponding to a missing follow‐up rate of 13.1% (Table S3). To determine whether gut microbial features provide prognostic information, we constructed random survival forest models using CAGs and microbial functional modules ranked by variable importance. A set of seven predictors yielded optimal model performance (Figure 1G), comprising CAD‐enriched CAGs and the aromatic amino acid metabolism module. These microbial features were integrated into an out‐of‐bag MACE score. Patients with higher microbial risk scores had a significantly increased risk of subsequent cardiac events (log‐rank *p* = 0.00059, Figure 1H, Figure S7A). Moreover, microbial predictors provided additional prognostic value when combined with conventional cardiovascular risk factors (Figure S7B,C).

Among the discriminatory microbial features, CAG35 and CAG15 were further prioritized because of their high prevalence in the CAD population. *Bacteroides thetaiotaomicron* CAD.MAG212, the dominant MAG in CAG 35, was enriched in patients who subsequently developed cardiac events. CAD patients in the higher quartile of *B. thetaiotaomicron* CAD.MAG212 had an increased risk of cardiac event (HR 1.82; 1.06–3.14; *p* = 0.032; Figure 1I). By contrast, CAG8 was significantly depleted in patients with CAD who experienced MACE, and a higher abundance of *Prevotella* sp., a component of CAG8, was associated with a reduced risk of cardiovascular events (HR, 0.57; 0.44–0.98; *p* = 0.047; Figure 1J). Iterative correlation analysis further indicated that CAG35 was a major contributor to variation in the tryptophan pathway, as this association was largely attenuated after the corresponding MAGs were removed from the analysis (Figure S7D, Table S10).

Given the prognostic relevance of microbiota‐associated aromatic amino acid metabolism, we next performed targeted metabolomic analysis. Baseline serum levels of phenylacetic acid and indole‐3‐carboxylic acid were predictive of subsequent MACE (Figure 1K,L). In patients without baseline MI, higher concentrations of phenylacetic acid and indole‐3‐carboxylic acid were also associated with increased MACE risk (Figure S8A). In contrast, circulating levels of the aromatic amino acid precursor tyrosine and the downstream indole‐3‐acetamide were lower in patients who experienced MACE than in those who did not (Figure S8B). While, fecal microbiota transplantation experiments showed that colonization with CAD‐associated microbiota induced higher fecal tyrosine levels (Figure S10C).

We further re‐constructed microbial genes involved in phenylalanine and tryptophan biosynthesis in *B. thetaiotaomicron* CAD.MAG212, and found significant enrichment of the *aat* and *fldH* genes in patients with CAD (Figure S9A,B). The positive correlation between *B. thetaiotaomicron* CAD.MAG212 abundance and serum phenylacetic acid levels among individuals who developed MACE further supports the potential importance of this bacterium and its associated aromatic amino acid metabolic capacity in cardiovascular event prediction (Figure 1M).

### TRANSMISSION OF ATHEROSCLEROSIS SUSCEPTIBILITY TO MICE VIA GUT MICROBIOTA TRANSPLANTATION

**Figure 2:**
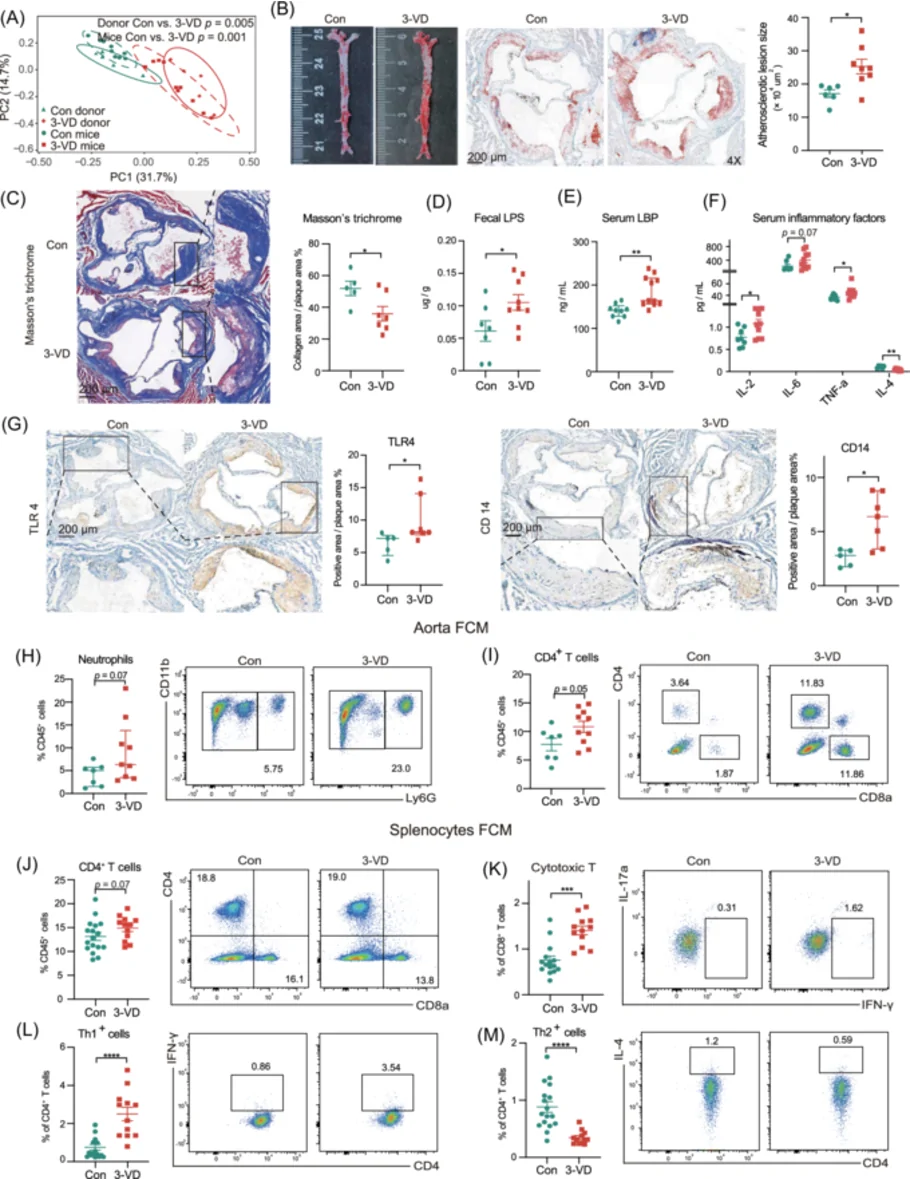
Fecal microbiota transplantation demonstrates that CAD‐associated gut microbiota promotes systemic and vascular inflammation and accelerates atherosclerotic lesion formation. (A) Principal coordinates analysis (PCoA) of the fecal microbiota from human donors with 3‐Vessel Disease (3‐VD) or control (Con) individuals and corresponding Abx‐treated SPF *ApoE* ^−/−^ mouse recipients. Differences in beta diversity were assessed using PerManova with 9999 permutations. (B) Representative Oil Red O‐staining of atherosclerotic plaques in en face aortas (*n* = 3 per group) and aortic root sections (*n* = 6–8 per group). The black bar represents 200 μm. (C) Representative Masson's trichrome staining of the aortic sinus showing collagen content within atherosclerotic plaques. Collagen area was quantified as a percentage of the total aortic sinus lesion area (*n* = 5–7 per group). (D) Fecal lipopolysaccharide (LPS) levels in Abx‐treated SPF *ApoE* ^−/−^ mice gavaged with fecal microbiota from the Con or 3‐VD donors (*n* = 7–9 per group). Serum levels of LPS‐binding protein (LBP) (E) and inflammatory cytokines (F) in mice receiving fecal microbiota from Con or 3‐VD donors (*n* = 9–11 per group). Cytokines include interleukin‐2 (IL‐2), IL‐6, tumor necrosis factor (TNF‐α) and IL‐4. (G) Representative immunostaining and quantitative analysis of Toll‐like receptor 4 (TLR4) and CD14 expression in the aortic sinus (*n* = 5–7 per group). (H) and (I) Flow‐cytometric analysis of neutrophils (CD11b^+^ Ly6G^+^) and CD4^+^ T cells in the aortas of germ‐free *ApoE* ^−/−^ mice gavaged with fecal microbiota from Con or 3‐VD donors (*n* = 6–10 per group). Cell populations are presented as percentages of CD45^+^ immune cells. (J) Flow‐cytometric analysis of CD4^+^ T cell populations in the spleen (*n* = 12–17 per group). (K) Representative flow‐cytometry plots and quantification of cytotoxic T cells, defined as CD8a^+^ IFN‐γ^+^ IL17a^−^ cells, in the spleen. (L) and (M) Representative flow‐cytometry plots and quantification of Th1^+^ (L) and Th2^+^ (M) cells within the splenic CD4^+^ T cell compartment (*n* = 12–17 per group). Normality was assessed by the Kolmogorov–Smirnov test, for (B), (C), (D), (F), and (H)–(M), data are presented as mean ± s.e.m. For (E) and (G), data are presented as median with interquartile range. Statistical significance was determined using a two‐tailed Student's *t*‐test or Mann–Whitney *U*‐test. *p < 0.05, **p < 0.01, ***p < 0.001, ****p < 0.0001.

To investigate whether CAD‐associated microbiota can contribute causally to atherosclerosis, fecal samples from control donors (*n* = 5) and patients with 3‐VD (*n* = 5) were transplanted into antibiotic‐treated specific pathogen‐free (SPF) *ApoE* ^−/−^ mice. After 20 weeks of colonization under a high‐fat diet, the gut microbiome profiles of recipient mice showed overall similarity to those of the corresponding human donor, indicating successful microbiota engraftment (Figure 2A). Notably, Oil Red O staining of whole aorta and aortic root sections exhibited markedly increased atherosclerotic lesion formation in mice colonized with microbiota from patients with 3‐VD (Figure 2B). Masson's trichrome staining further indicated features consistent with reduced plaque stability in these mice (Figure 2C). In parallel, fecal LPS levels and serum LBP concentrations were elevated in the 3‐VD microbiota‐recipient mice, mirroring the patterns observed in human donors (Figure 2D,E). Circulating proatherogenic cytokines, including interleukin‐2 (IL‐2), IL‐6, and tumor necrosis factor (TNF‐α), were also increased in the 3‐VD group (Figure 2F).

Consistent with enhanced endotoxin‐associated inflammatory signaling, vascular expression of Toll‐like receptor 4 (TLR4), the principal pattern‐recognition receptor for LPS, and its co‐receptor CD14 [16] was significantly elevated in mice receiving 3‐VD microbiota (Figure 2G). Downstream proatherogenic mediators, including monocyte chemoattractant protein‐1 (MCP‐1) and matrix metalloproteinase‐9 (MMP‐9), were similarly increased (Figure S10B). Together, these findings demonstrate that CAD‐associated microbiota can transmit susceptibility to accelerated atherosclerosis in *ApoE* ^−/−^ mice, potentially through enhanced LPS production and activation of vascular inflammatory pathways.

### CAD‐ASSOCIATED MICROBIOTA EXACERBATE VASCULAR INFLAMMATION AND SYSTEMIC TH1/TH2 IMBALANCE

We next performed transcriptome profiling of aortic tissues to define the immune programs activated by CAD‐associated microbiota. Gene‐expression analysis revealed enrichment of pathways related to T‐cell activation and leukocyte proliferation in 3‐VD mice (Figure S10D). To further dissect these immune alterations, pooled fecal microbiota from control individuals and patients with 3‐VD were transplanted into germ‐free *ApoE* ^−/−^ mice.

Flow‐cytometric analysis (Figure S11) showed that mice receiving 3‐VD microbiota showed an increasing trend of CD4^+^ T cells and neutrophils in the aorta compared with mice receiving the control microbiota (Figure 2H,I, Figure S12B). Given that LPS can modulate CD4^+^ T cell responses, especially Th1 polarization [17], we further examined systemic T‐cell subsets. Mice colonized with 3‐VD microbiota exhibited expansion of CD4^+^ T cells and pronounced immune activation, characterized by elevated Th1 cells (CD4^+^ CD8a^−^ IFN‐γ^+^) and reduced Th2 cells (CD4^+^ CD8a^−^ IL4^+^) in the spleen (Figure 2J,L,M). In addition, effector cytotoxic T cell activity was increased in the 3‐VD group (Figure 2K).

These results suggest that CAD‐associated microbiota promote vascular immune activation and skew systemic adaptive immunity toward a proatherogenic Th1‐dominant state (Figure S12C). By contrast, no significant difference in the Th17/Treg ratio was observed (Figure S12D). Collectively, these results support a causal role for CAD‐associated gut microbiota in enhancing vascular inflammation and accelerating the immunopathological process underlying atherosclerosis.

## POTENTIAL APPLICATION AND LIMITATIONS

This study highlighted the need to elucidate CAD stage‐specific microbial profiles that may be useful for modeling disease progression and demonstrated the transmission of atherosclerosis susceptibility through gut microbiota transplantation. By integrating longitudinal follow‐up with multi‐omics profiling and experimental validation, we extend previous cross‐sectional observations and link disrupted microbial homeostasis to cardiovascular risk.

Microbial co‐abundance networks changed progressively with increasing CAD severity and were accompanied by distinct metabolic alterations. These disease‐associated microbial profiles reflected functional alterations relevant to CAD pathophysiology. In particular, *Bacteroides spp*. were significantly enriched across CAD stages, consistent with their capacity to produce LPS [18], and may contribute to MACE risk through both endotoxin‐mediated inflammation and aromatic amino acid metabolism.

To explore causality, we transplanted fecal microbiota from patients with CAD into mice and showed that CAD‐associated microbiota accelerated atherosclerosis. This phenotype was accompanied by enhanced vascular immune activation, including increased neutrophil recruitment and CD4^+^ T cell activation. Furthermore, microbial LPS‐associated signaling appeared to skew systemic immunity toward a Th1‐dominant state, a process implicated in the initiation and progression of atherosclerosis.

Our study also establishes a microbiome‐based framework for cardiovascular event prediction. Although the prognostic model showed modest discriminatory performance, combining microbial features with conventional cardiovascular risk factors may improve early risk stratification for MACE. These findings are consistent with emerging evidence linking gut microbiota and microbial metabolites to arterial thrombosis [19], as well as our complementary work showing that the gut commensal *Bacteroides thetaiotaomicron* promotes atherothrombosis by regulating tryptophan metabolism [20].

Several limitations should be acknowledged. First, validation in larger, independent, and multicentre cohorts is required to assess the generalizability of these findings. Second, residual confounding by factors, such as age and smoking, cannot be fully excluded, despite extensive clinical annotation. Third, the effects of drugs on the gut microbiome could not be systematically evaluated because most patients received guideline‐directed therapy. Fourth, the relatively limited follow‐up duration precluded robust subgroup analyses of specific hard endpoints, such as cardiac death. Fifth, the SPF and germ‐free *ApoE* ^−/−^ FMT experiments differed in sex composition (all male vs. sex‐matched, respectively), which may have contributed to the immune phenotypes observed and should be examined in future sex‐stratified studies. Finally, although fecal microbiota transplantation experiments support a causal contribution of CAD‐associated microbiota to atherosclerosis, the mechanistic chain connecting specific microbial taxa, metabolites, immune pathways, and cardiovascular events requires further investigation.

Identifying microbiome‐derived prognostic biomarkers may facilitate the development of non‐invasive diagnostic tools that complement existing cardiovascular screening and prevention strategies. Collectively, our findings provide a translational framework for mapping microbiome‐host interactions across CAD stages.

## Supporting information


**Figure S1:** Distribution of Gensini and Syntax score representing atherosclerosis severity in each subgroup of coronary artery disease (CAD).
**Figure S2:** The pipeline of metagenomic sequencing data analysis.
**Figure S3:** MAG‐level network diagram showing the enrichments of MAGs in Con group.
**Figure S4:** Correlation profile of fasting serum metabotypes and physiological traits in partial 152 subjects.
**Figure S5:** LBP and LPS levels in the serum of CAD cohort.
**Figure S6:** Fine‐grained correlation profile of microbial functional modules, serum metabotypes, and clinical physiological traits.
**Figure S7:** Supplementary data from random survival forest for identifying microbial prognostic features for major adverse cardiac events (MACE).
**Figure S8:** Aromatic amino acids abundance test and Kaplan–Meier analysis.
**Figure S9:** Partial microbial‐dependent aromatic amino acids pathways and key microbial genes abundance test.
**Figure S10:** Supplementary experimental data of fecal microbiota transplantation experiment.
**Figure S11:** Gating strategy for the flow cytometric analyses and schematic of the screening procedure.
**Figure S12:** Supplementary flow cytometry data from fecal microbiota transplantation of germ free *ApoE* ^−/−^ mice.

**Table S1:** Clinical characteristics of CAD cohort at baseline.
**Table S2:** Supplementary clinical characteristics including dietary intake, Bristol score, and physical exercise in CAD cohort.
**Table S3:** Follow‐up outcomes of CAD cohort.
**Table S4:** Statistics for the metagenomic shotgun sequencing data of 319 individuals.
**Table S5:** The assembly results of metagenome‐assembled genomes (MAGs).
**Table S6:** Univariable PerManova/Adonis analysis of host factors associated with MAG‐level Bray–Curtis dissimilarity.
**Table S7:** Co‐abundance groups (CAGs) and the list of MAGs in each cluster.
**Table S8:** Description of serum metabotypes and their associations with CAD severity phenotypes.
**Table S9:** Associations between gut microbial functional modules and CAD severity phenotypes.
**Table S10:** Main microbial driver CAGs for the associations between microbial functional modules and CAD severity phenotypes.
**Table S11:** Strengthening The Organization and Reporting of Microbiome Studies (STORMS) checklist.

## Data Availability

The raw sequence data reported in this paper have been deposited in the Genome Sequence Archive in the National Genomics Data Center, China National Center for Bioinformation/Beijing Institute of Genomics, Chinese Academy of Sciences (GSA: HRA010791) https://ngdc.cncb.ac.cn/gsa-human/browse/HRA010791. Due to ethical and legal restrictions, deidentified individual participant data and the data dictionary cannot be made publicly available. All data are available upon request to the corresponding author and subject to local rules and regulations. The data and scripts used in this study can be found on GitHub: (https://github.com/LiangLifeng/CHD-iMeta-2026). STORMS (Strengthening The Organization and Reporting of Microbiome Studies, Table S11) checklist was provided for concise and complete reporting of human microbiome study. Supplementary materials (methods, figures, tables, graphical abstract, slides, videos, Chinese translated version, and updated materials) may be found in the online DOI or iMeta Science http://www.imeta.science/.
