# Supplementary material for: Gut microbiota‐mediated immune and metabolic dysregulation in coronary artery disease progression and prognosis
Source: Imeta. 2026 Jul 1;5(4):e70147. doi: 10.1002/imt2.70147 (PMC13519092; doi:10.1002/imt2.70147)
Supplement: Supplementary file 1 [file IMT2-5-e70147-s001.pdf]

Supporting information to

Gut microbiota-mediated immune and metabolic dysregulation in coronary artery disease progression and prognosis

Running title: Gut microbiota and coronary artery disease

Honghong Liu[1,2,3#], Siqin Feng[1,3#], Lifeng Liang[4#], Muyun Tang[2,3], Yifei Wang[2], Yiyang Wang[2], Zhiyu Zhang[2], Haiping Xing[1,2], Chenhong Zhang[5,6*], Ran Tian[2*], Shuyang Zhang[2,3*]

[1] Department of Internal medicine, Peking Union Medical College Hospital, Chinese Academy of Medical Sciences & Peking Union Medical College, Beijing, 100730, China

[2] Department of Cardiology, Peking Union Medical College Hospital, Chinese Academy of Medical Sciences & Peking Union Medical College, Beijing, 100730, China

[3] State Key Laboratory of Complex Severe and Rare Diseases, Peking Union Medical College Hospital, Chinese Academy of Medical Sciences & Peking Union Medical College, Beijing, 100730, China

[4] 01Life Institute, Shenzhen, 518000, China

[5] State Key Laboratory of Microbial Metabolism, School of Life Sciences and Biotechnology, Shanghai Jiao Tong University, Shanghai, 200240, China

[6] Joint International Research Laboratory of Metabolic & Developmental Science, Shanghai Jiao Tong University, Shanghai, 200240, China

[#] These authors contributed equally: Honghong Liu, Siqin Feng, Lifeng Liang

[*]Correspondence: zhangchenhong@sjtu.edu.cn (Chenhong Zhang), ron_tian@163.com (Ran Tian) and shuyangzhang103@nrdrs.org (Shuyang Zhang)

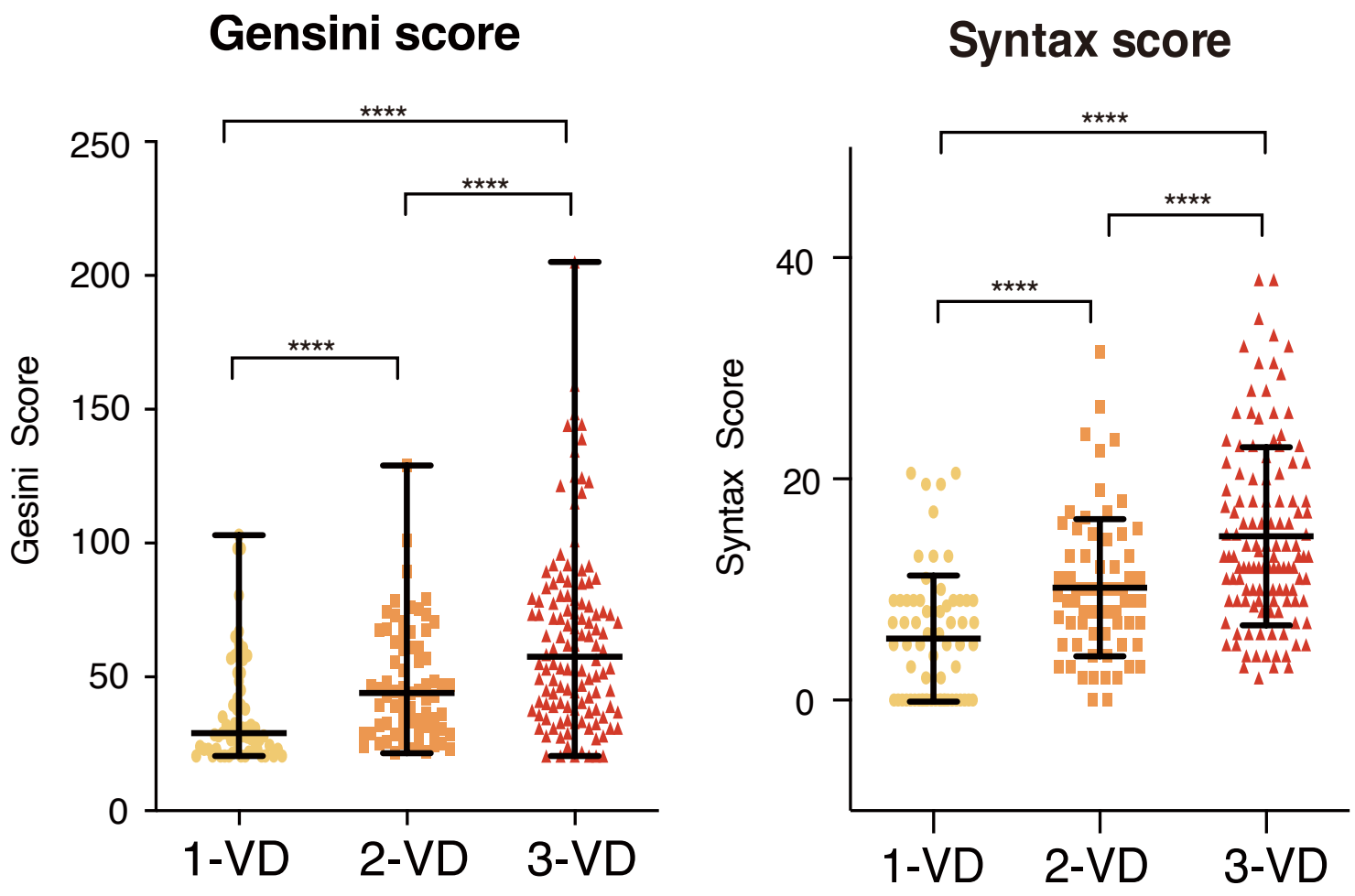

Figure S1. Distribution of Gensini and Syntax score representing atherosclerosis severity in each subgroup of coronary artery disease (CAD). Group-level abundance differentiation of Gensini and Syntax scores representing atherosclerosis severity. Data are visualized by a box plot. The box represents the interquartile range. The line inside the box represents the median. $^{****}p$ value < 0.0001.

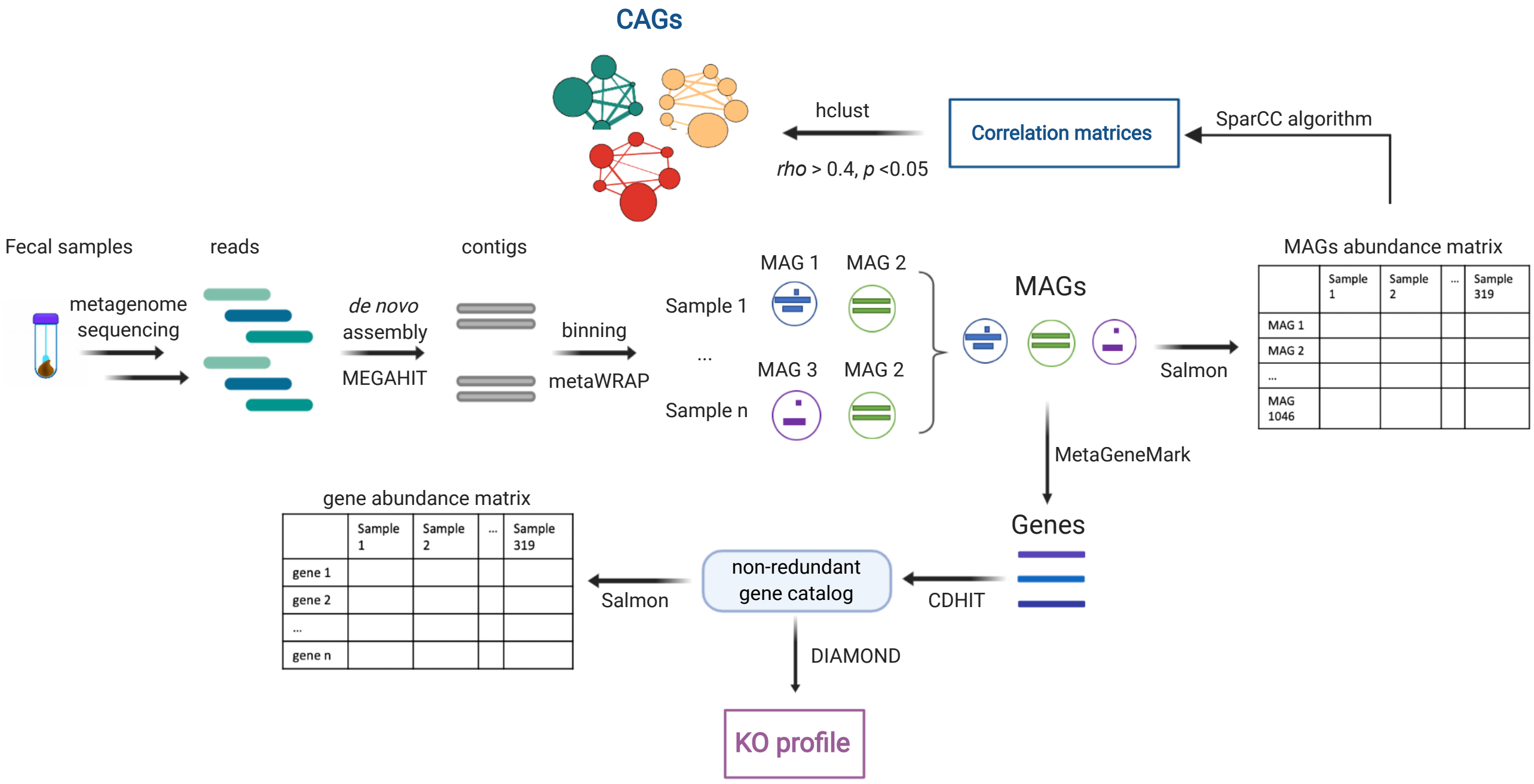

Figure S2. The pipeline of metagenomic sequencing data analysis. CAG, co-abundant groups; MAG, metagenome-assembled genomes.

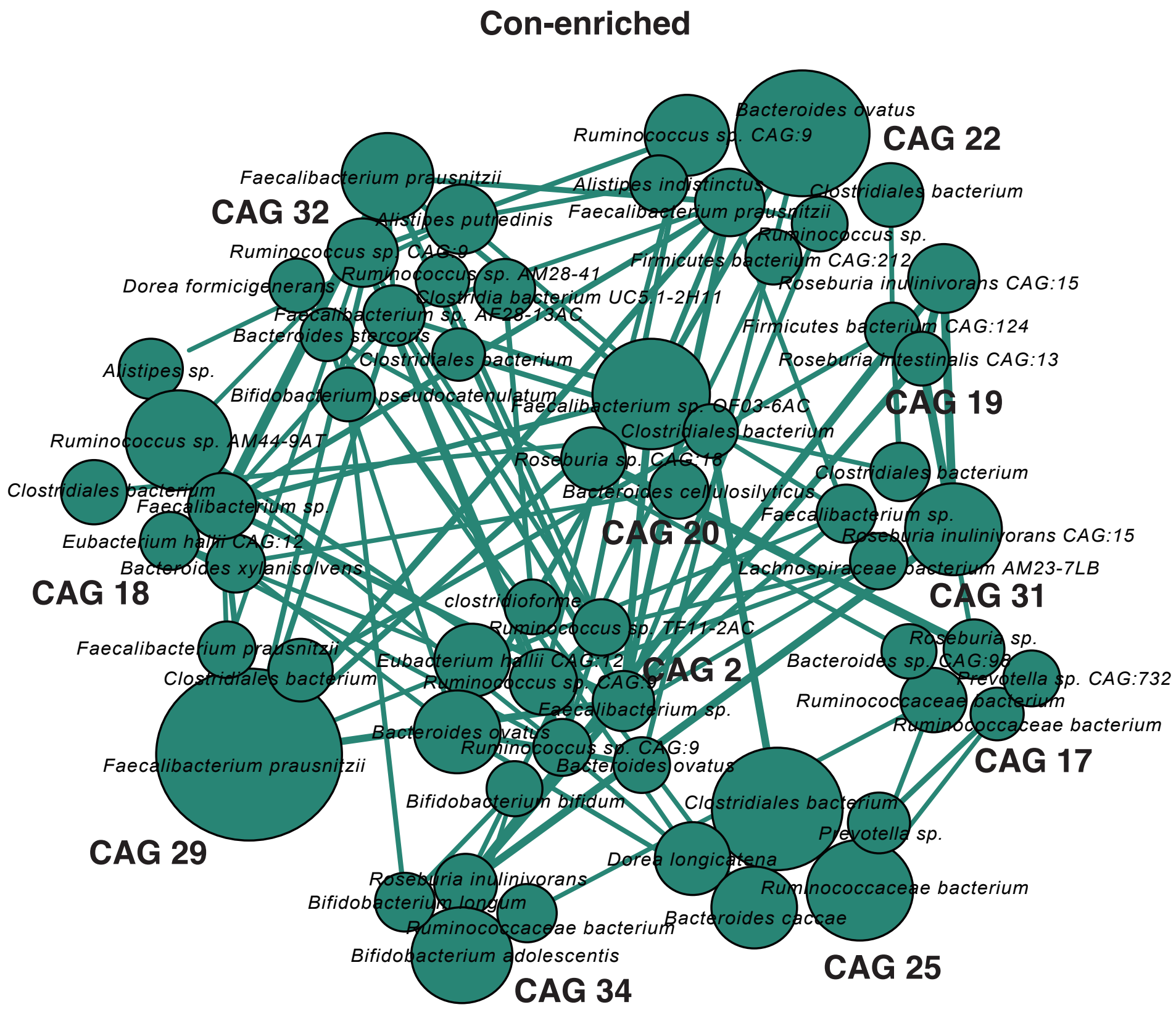

Figure S3. MAG-level network diagram showing the enrichments of MAGs in Con group. Node size indicates the mean abundance of each MAG. The bacteria denoted on the nodes were of the lowest classification status that could be clearly identified. Lines between nodes represent correlations between the corresponding nodes. Only lines corresponding to correlations with magnitudes greater than 0.4 were drawn.

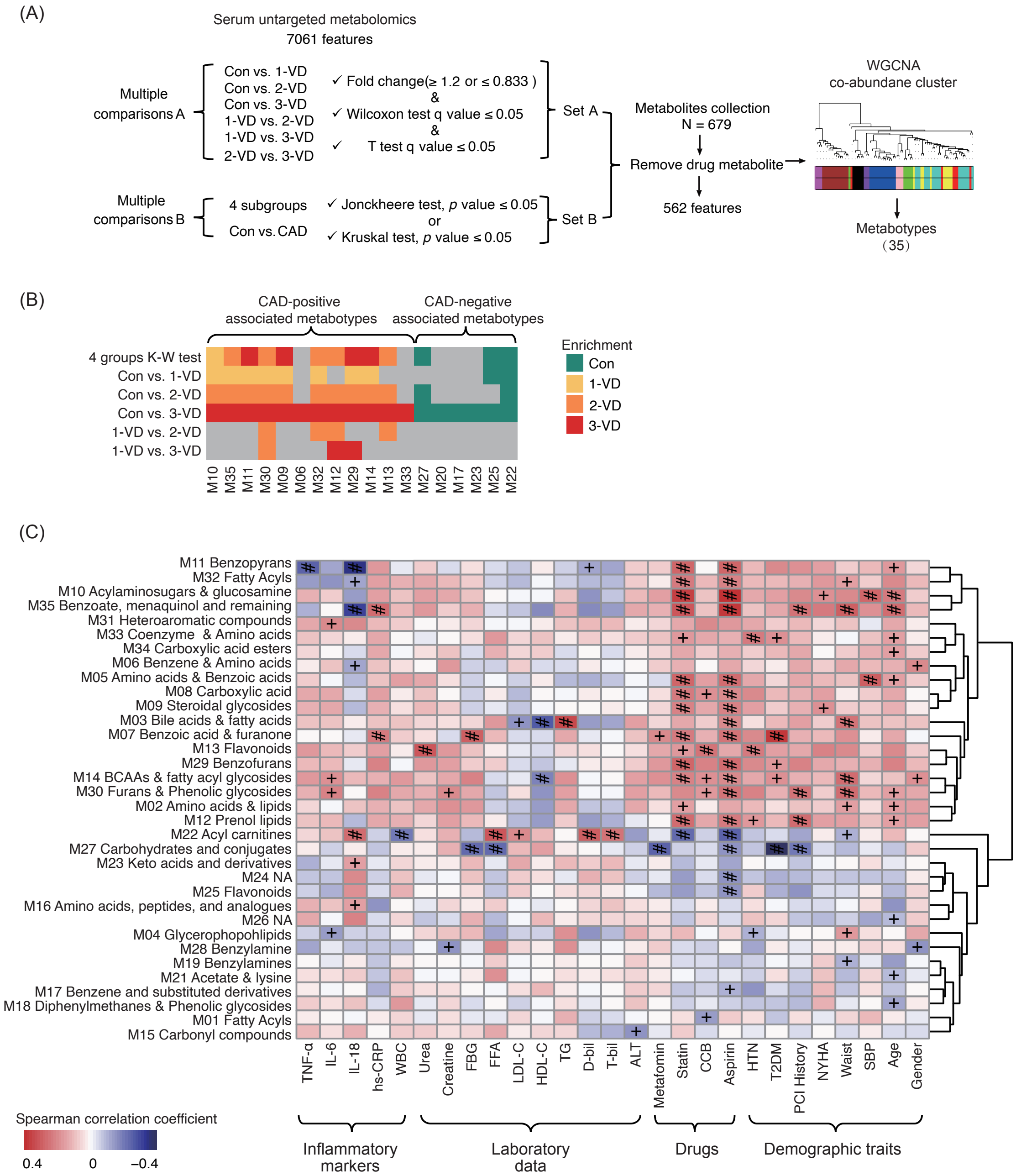

Figure S4. Profile of fasting serum metabolite metabotypes and physiological traits in 152 subjects. (A) We conducted a "cross-comparison scheme" to identify the serum metabolome features in different CAD groups. Serum metabolites were summarized as co-abundance metabolic modules, and disturbed features were filtered for significant positive or negative associations with CAD phenotype. (B) Serum metabolite modules significantly changed between different CAD groups according to the corresponding test. (C) Spearman correlations between serum metabotypes and clinical phenotypes. Statistical significance after FDR correction was denoted: + $q < 0.1$, # $q < 0.01$. Con, controls; 1-VD, 1-vessel disease; 2-VD, 2-vessel disease; 3-VD, 3-vessel disease. TNF-α, tumor necrosis factor-alpha; IL-6, interleukin -6; IL-18, interleukin-18; hs-CRP, high-sensitivity C-reactive protein; WBC, white blood cells; FBG, fasting blood glucose; LDL-C, low-density lipoprotein cholesterol; HDL-C, high-density lipoprotein cholesterol; TG, triglyceride; T-biL, total bilirubin; ALT, alanine aminotransferase; CCB, calcium channel blockers; HTN, hypertension; T2DM, type 2 diabetes mellitus; SBP, systolic blood pressure.

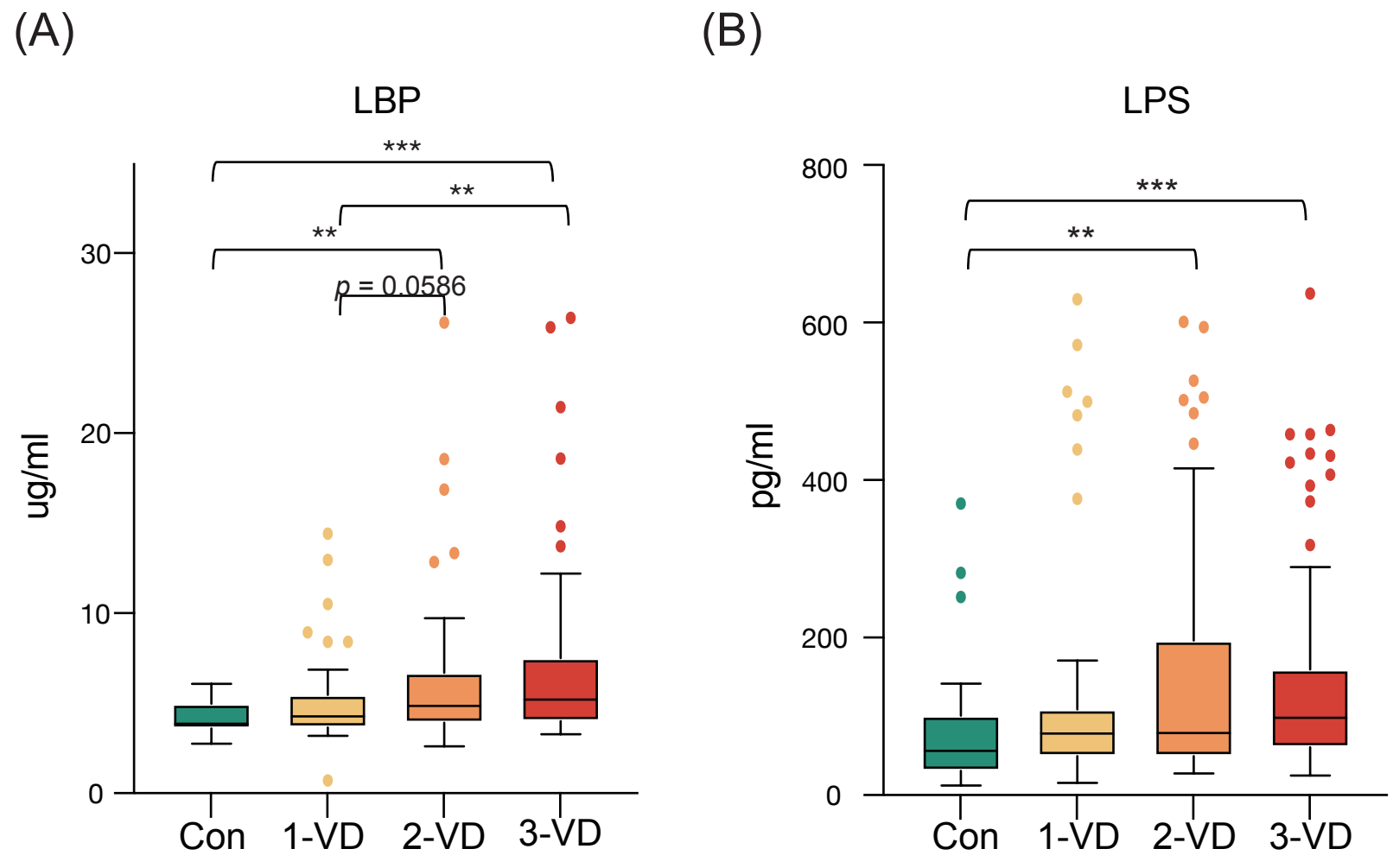

Figure S5. LPS-binding protein (LBP) and lipopolysaccharide (LPS) level in the serum of CAD cohort. Data are visualized by a box plot. The box represents the interquartile range. The line inside the box represents the median. ** p value < 0.01, *** p value < 0.001.

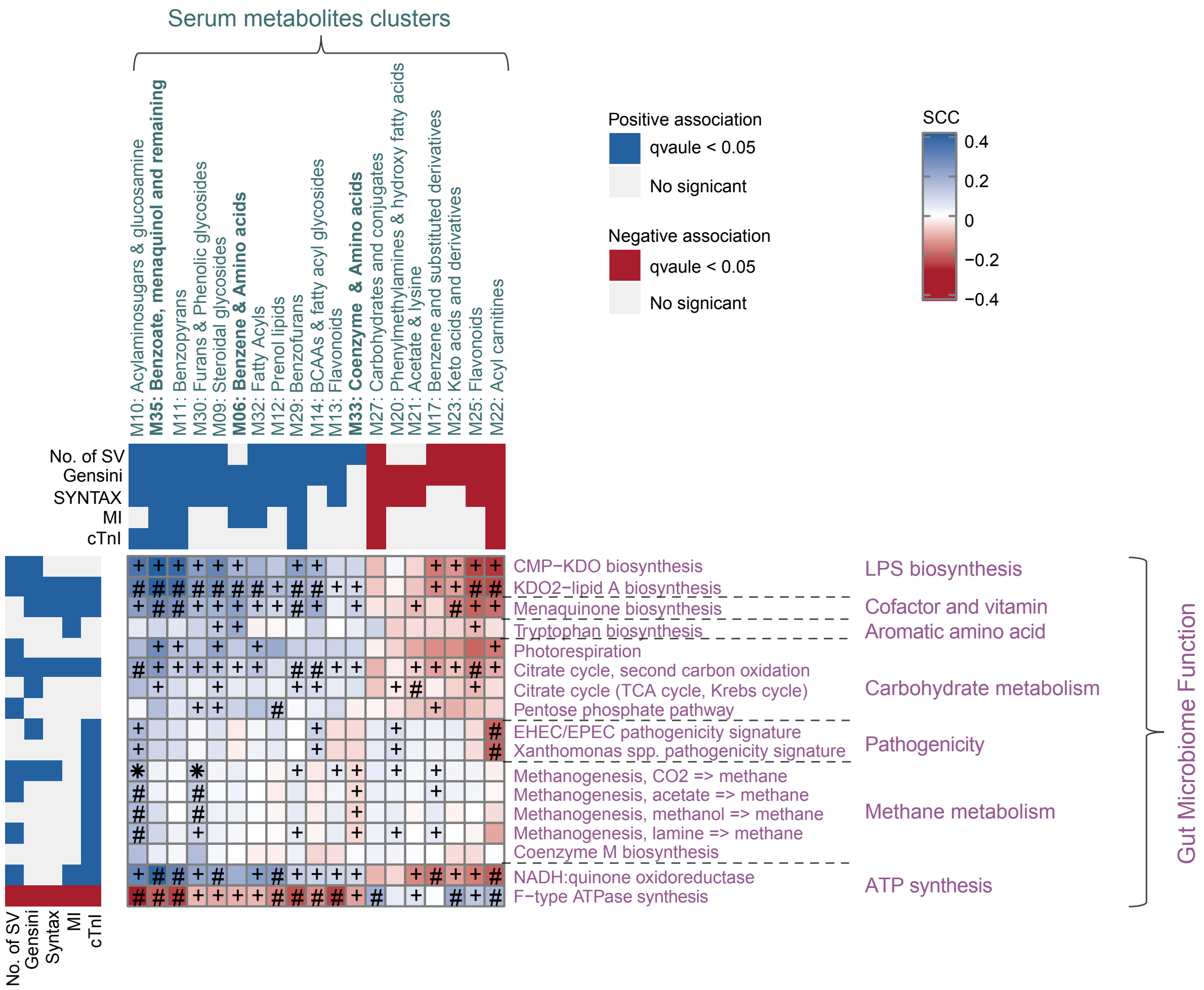

Figure S6. Fine-grained correlation profile of microbial functional modules, serum metabotypes and clinical physiological traits. The left and upper panels show significant associations between either microbial functional modules or serum metabotypes and clinical phenotypes; coloring indicates the direction of the association. The middle panel shows associations between the same modules and serum metabotypes. Coloring represents the median Spearman correlation coefficient, + $q < 0.1$, # $q < 0.01$, * $q < 0.001$. No. of SV, Number of stenosed vessels; MI, myocardial infarction; cTnI, troponin I; SCC, Spearman's rank correlation coefficient.

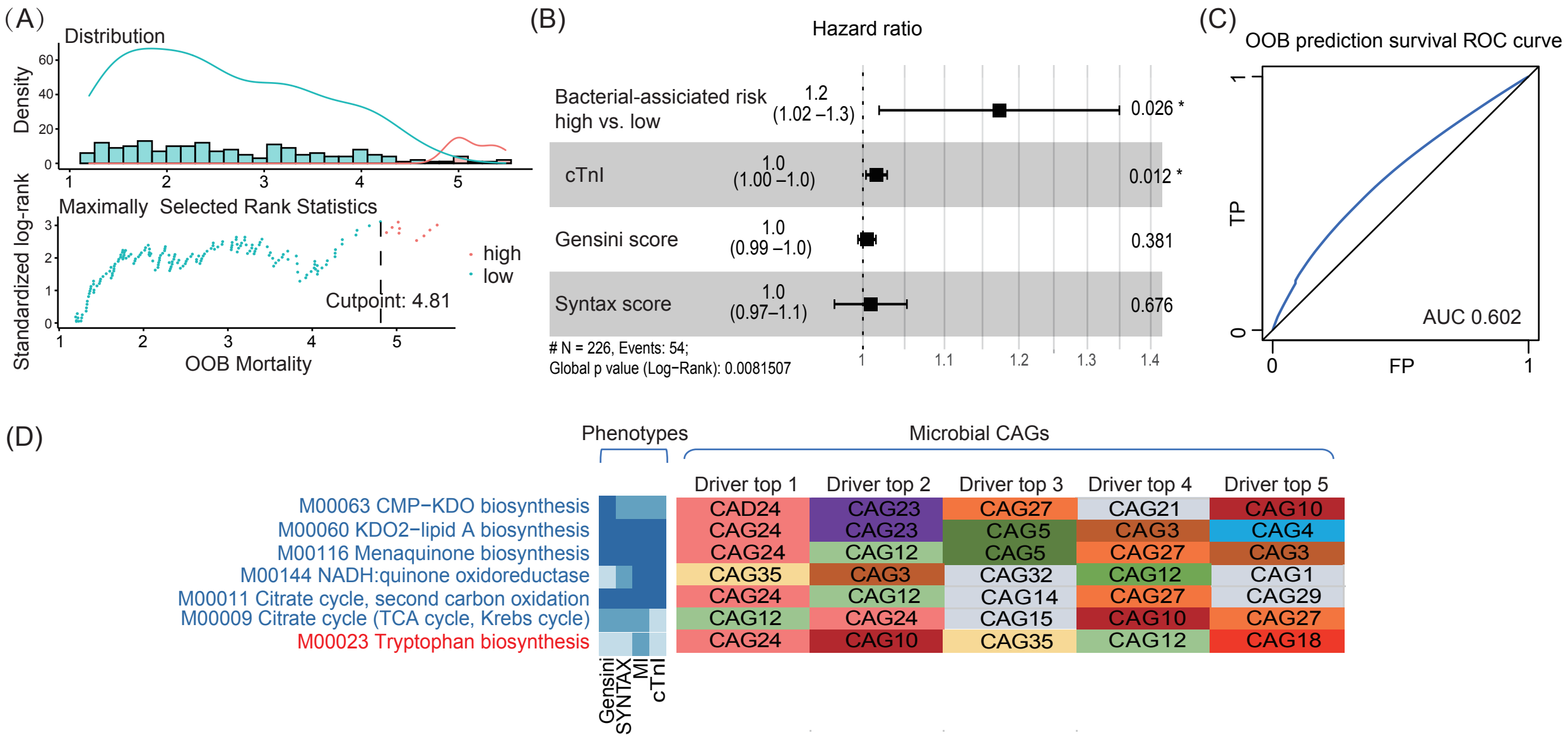

Figure S7. Supplementary data from random survival forest for identifying microbial prognostic features for cardiovascular outcomes. (A) Determining the appropriate cut-off value to categorize the OOB (out of bag) -score (high vs. low). (B) Forest plot summary of multivariate Cox regression analysis of bacterial-associated risk groups and baseline clinical covariates. Hazard ratios are visualized with 95% CIs. (C) CVD outcomes prognosis accuracy is shown via receiver operating characteristic (ROC) curves. (D) The five most important microbial CAGs driving the association between the indicated microbiome functional modules and CAD severity phenotypes are shown. Each CAG is highlighted with a different colour. The left sidebar represents positive (blue) associations between the functional modules and the indicated phenotypes (q value < 0.1).

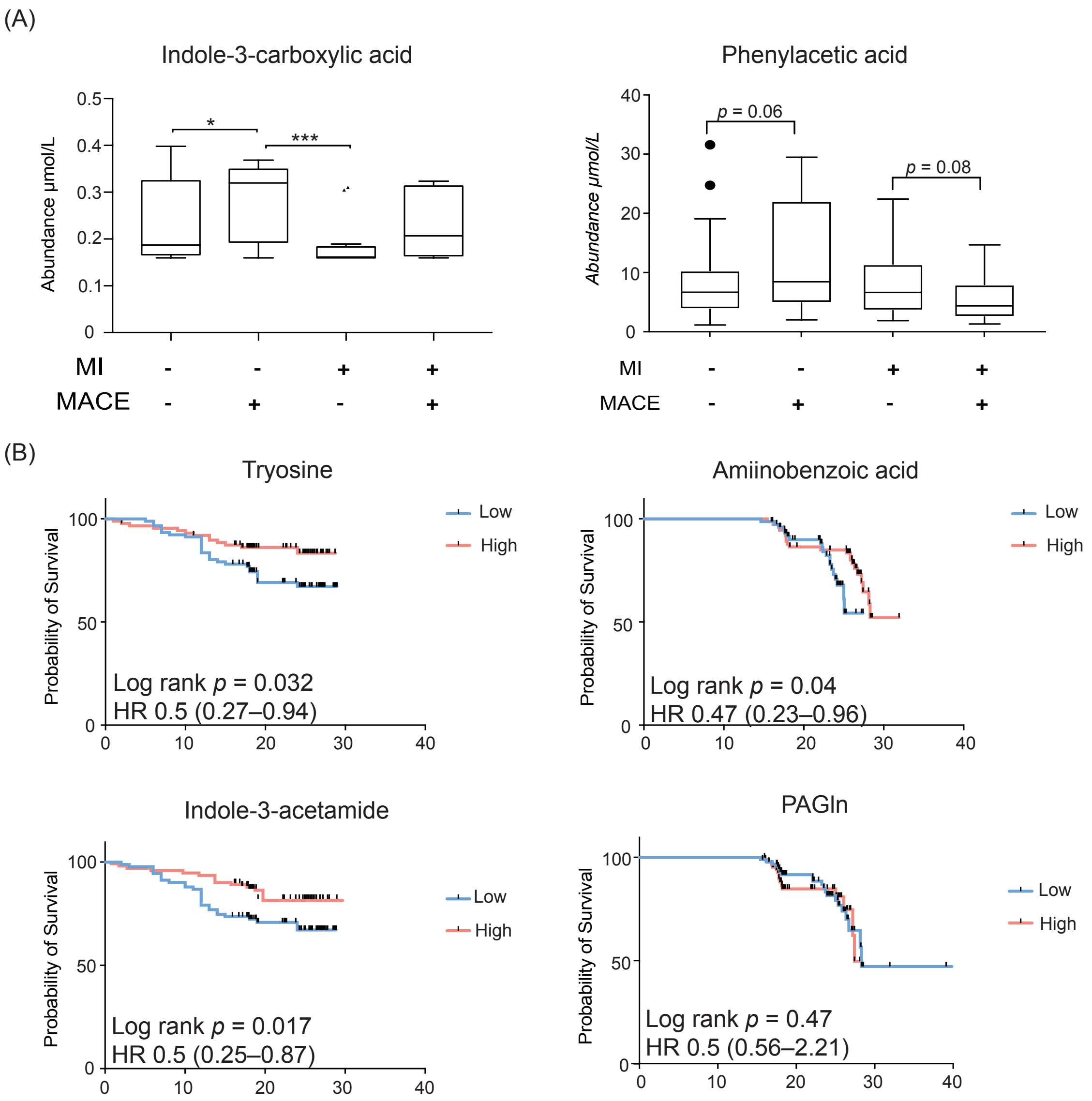

Figure S8. Aromatic amino acids abundance test and Kaplan–Meier analysis. (A) Relative abundance of indole-3-carboxylic acid or phenylacetic acid in myocardial infarction patients with major adverse cardiac event (MACE) and without. (B) Kaplan–Meier estimates of outcomes according to the quartile (high vs. low) of other aromatic amino acids. PAGln, phenylacetylglutamine.

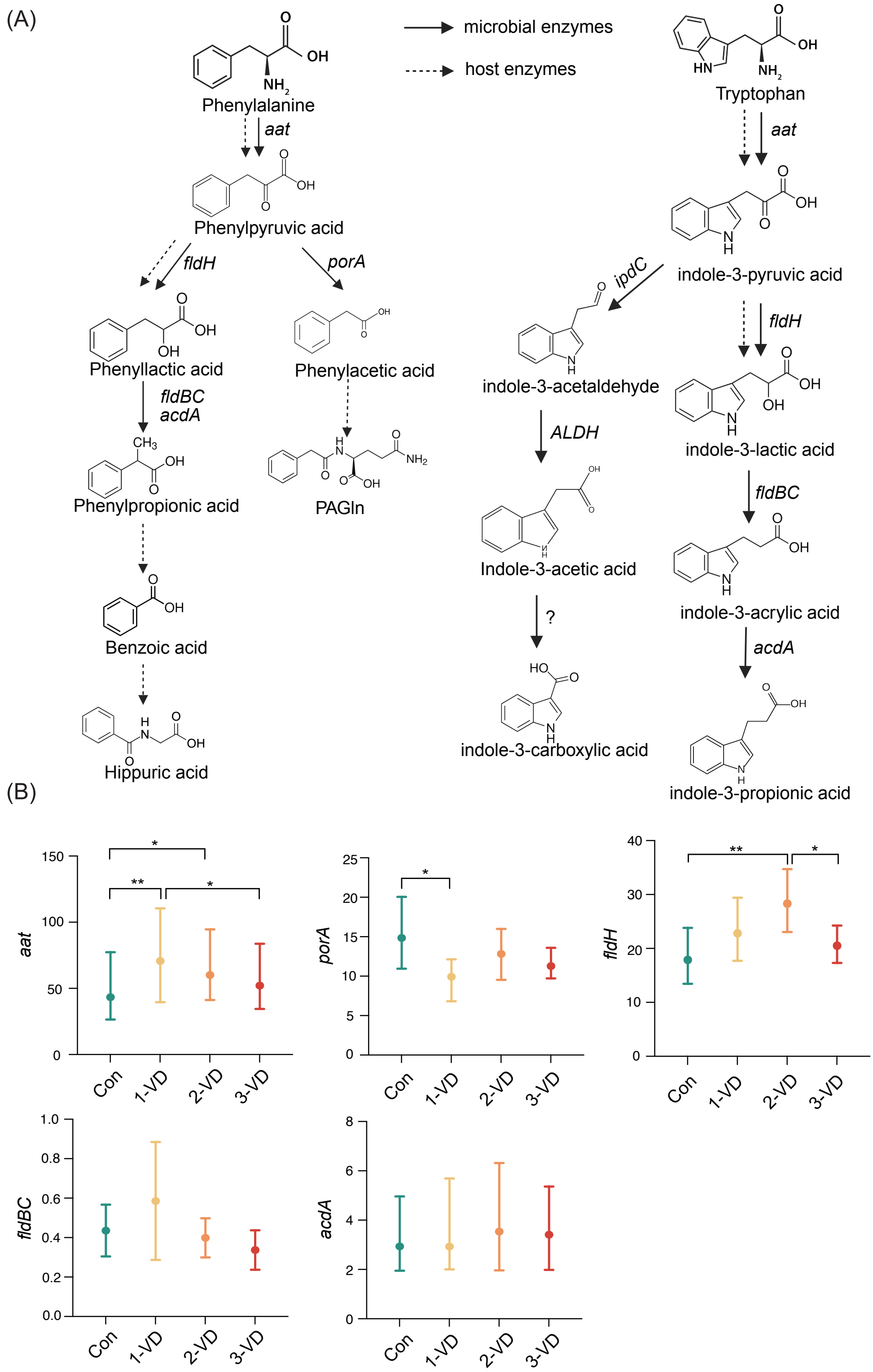

Figure S9. Partial microbial Phenylalanine and Trptophan pathways and key KO genes abundance test. **(A)** Pathways involved in the metabolism of certain aromatic amino acids. (B) Relative abundance of KO genes involved in aromatic amino acids expressed in *Bacteroides thetaiotaomicron CAD. MAG212*. showed difference between different groups at baseline. M-W test, *$p < 0.05$, **$p < 0.01$. PAGln, phenylacetylglutamine.

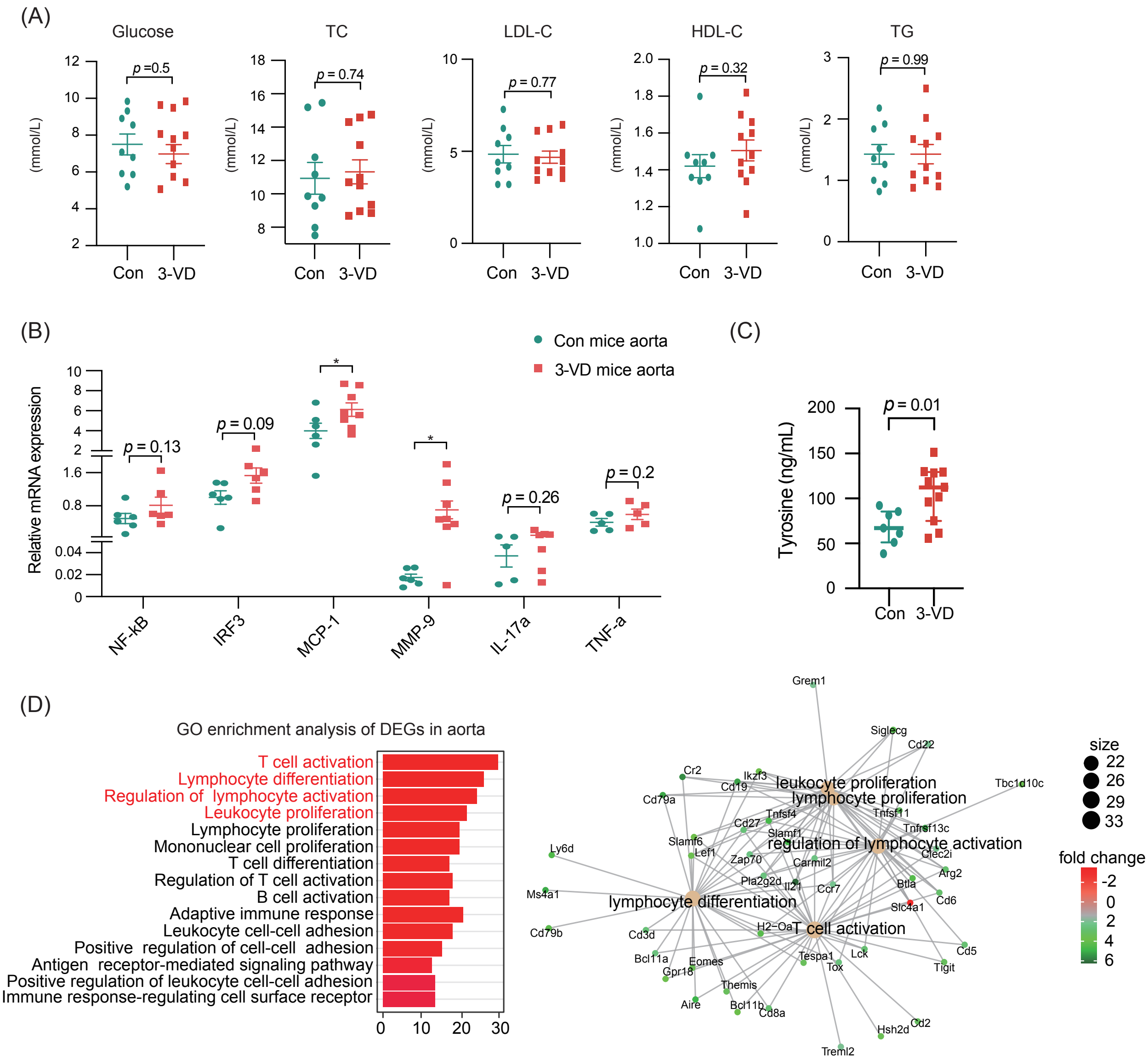

Figure S10. Supplementary experimental data from fecal microbiota translation of Con and CAD donors. (A) Serum glucose, total cholesterol (TC), low density lipoprotein-cholesterol (LDL-C), high density lipoprotein-cholesterol (HDL-C) and triglyceride (TG) levels of Con and 3-VD gavaged *ApoE* $^{-/-}$ mice. (B) mRNA levels of inflammatory signals in the atherosclerotic aorta. Data were normalized to the housekeeping *Gapdh* gene. NF-κB, nuclear factor kappa-light-chain-enhancer of activated B cells; IRF3, interferon regulatory factor 3; MCP-1, monocyte chemoattractant protein-1; MMP-9, matrix metalloproteinase-9; IL-17a, interleukin-17a; TNF-α, tumor necrosis factor-α. (C) Fecal level of tyrosine in Con and 3-VD gavaged *ApoE* $^{-/-}$ mice. (D) Results of differentially expressed gene ortholog (GO) enrichment and KEGG enrichment in the aorta, respectively. For B, C and D, data are presented as median with IQR. p values were determined by the Mann–Whitney U-test. * $p < 0.05$.

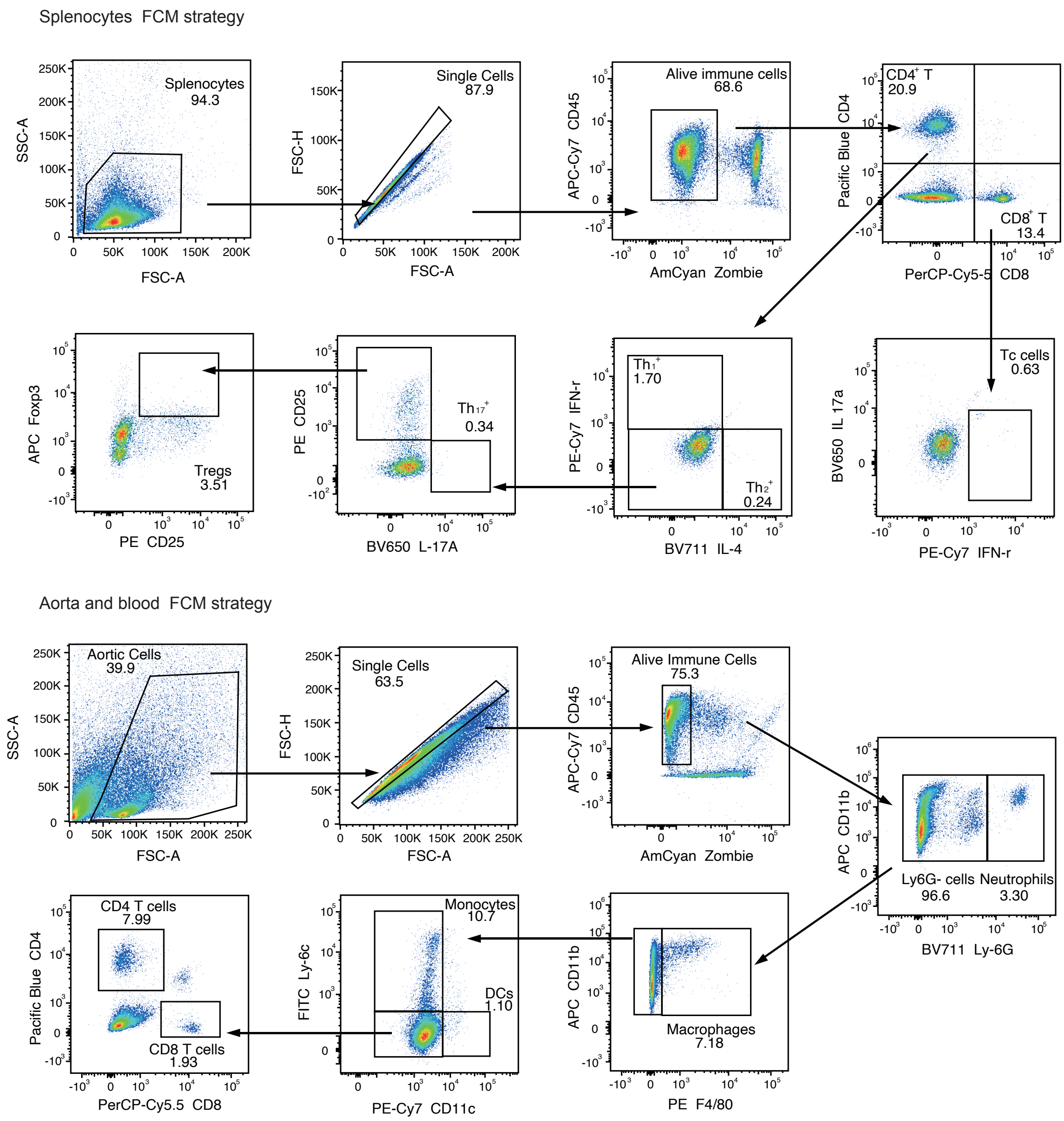

Figure S11. Gating strategy for the flow cytometric (FCM) analyses.

Splenocytes were isolated and stained with fluorescently labeled antibodies. Cells were first gated on singlets, followed by selection of live cells using a viability dye. Lymphocytes were then identified defined as $CD45^{+}$. Subsequent sequential gating was performed to identify specific immune cell subsets. For aortic and blood cells, $CD45^{+}$ immune cells were identified using the same standardized gating strategy as applied to splenocytes. Within this gate, further discrimination of $Ly6G^{+}$ neutrophils from $Ly6G^{-}$ monocytes/macrophages was performed. Then macrophages were identified by F4/80 expression, and dendritic cells (DCs) were identified by co-expression of CD11c, while classical monocytes were characterized by high Ly6C.

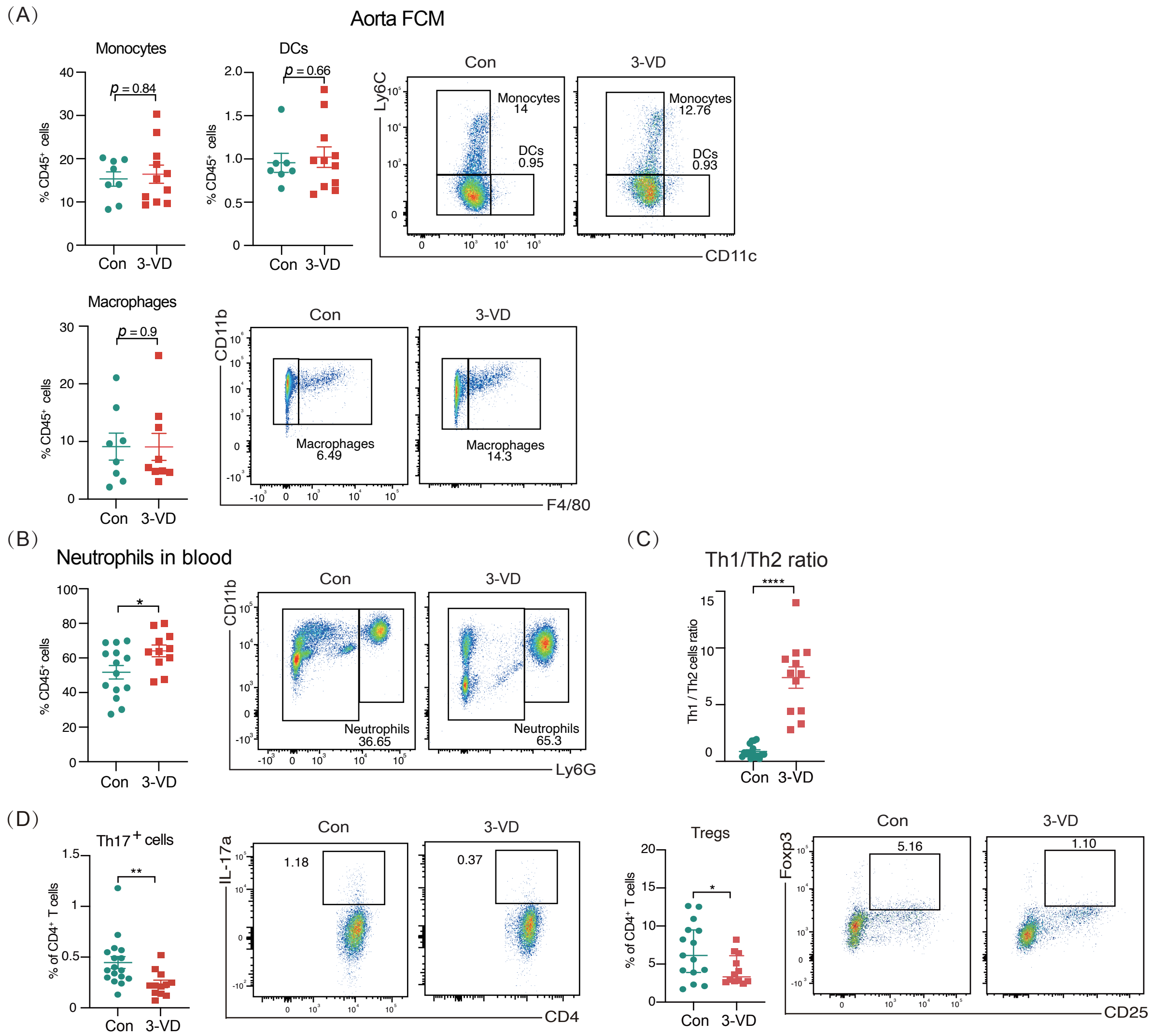

Figure S12. Supplementary flow cytometry data from fecal microbiota transplantation of GF *ApoE* $^{-/-}$ mice. (A) Monocytes (CD11b$^-$ CD11c$^-$ Ly6C$^+$), dendritic cells (DC, CD11c$^+$) and macrophages (CD11b$^+$ F4/80$^+$) distributions of Con and 3-VD of GF *ApoE* $^{-/-}$ aorta gavged with faeces from Con or 3-VD donors. (B) Neutrophils (CD11b$^+$ Ly6G$^+$) percentages in blood. (C) Th1/Th2 ratio in spleen. (D) Th17$^+$ (IL17a$^+$ CD25$^-$) and Tregs (CD25$^+$ Foxp3$^+$) expressed in spleen. For all flow cytometric results, data are presented as means ± s.e.m.. *p* values were determined by two-tailed Student's t-test, * $p < 0.05$, ** $p < 0.01$.

Methods

Study populations

Baseline study design and populations

This metagenomics CAD cohort was a single-center, prospective study approved by the Peking Union Medical College Hospital Board. The study was approved by local ethics committees (JS-1195, Peking Union Medical College Hospital, Beijing), and informed consent was obtained from all subjects. Sequential consenting adult subjects (ages 45 years and above) who mainly came from north of China and were hospitalized for coronary angiography were eligible between 2016 and 2018. Obstructive coronary artery disease was defined as the presence of at least 1 major epicardial vessel with ≥ 50% stenosis [1]. Coronary atherosclerotic burden was calculated on the basis of angiographic disease of their native vessels before revascularization using the Gensini [2]and Syntax [3] score by two professional cardiologists in a blind. Severity of CAD was also quantified by the number of major epicardial coronary arteries with ≥ 50% stenosis. Patients who met CAD diagnostic criteria after coronary arteriography were further divided into single-vessel stenosed disease (1-VD, N = 66), double-vessel stenosed disease (2-VD, N = 71) and three-vessel stenosed disease (3-VD, N = 123) subgroups. Due to our study design, the sample size of CAD subgroups was slightly uneven. For controls, we enrolled subjects who exhibited negative results upon coronary artery CT or coronary angiography examination or were identified as having no CAD-related clinical signs and symptoms by cardiologists. For controls, we tried to select subjects from north of China so that their dietary habits would not differ much from those of CAD participants. For Control subjects, the

medication usage was recorded in the form of questionnaires, one subject was treated with long-term oral statin therapy, and no subjects reported long-term pain control medication. Subjects were excluded if they had gastrointestinal diseases (including inflammatory bowel disease, pancreatitis, chronic diarrhea), malignant active tumors, autoimmune disorders, patients with a history of organ transplantation or receiving immunosuppressive therapy, infectious diseases (including hepatitis B or C and human immunodeficiency viruses), renal dysfunction (severe renal disease creatinine > 3.0 mg/dl), a history of gastrointestinal surgery in the previous year or were administered antibiotics for more than 3 days in the previous 3 months.

Metadata collection

Baseline metadata covered participants' anthropometric features and information related to health status, disease history, medication, gastrointestinal conditions, dietary habits, sleep situation, and physical activity were collected with standardized questionnaires. The questionnaire is voluntary and only partial subjects are willing to take the questionnaire (Table S2). Current cigarette smoking was self-reported by participants. Drinking history was defined as drinking ≥ 50 g of alcohol per day. Body weight and height were measured with the subject wearing light indoor clothes without shoes, and body mass index was calculated as kilograms per meters squared. Waist circumference was measured at 5 cm above the navel at minimal respiration. Blood pressure (BP) was measured in the sitting position with automated electronic BP monitors by trained observers. BP readings were taken on each occasion at least twice with a 30–to–60s interval after 5 min of rest. Diabetes mellitus was defined as having a fasting

glucose ≥ 126 mg/dL or self-reported current treatment with antidiabetic medication (insulin or oral hypoglycemic agents).

For the participants, peripheral venous blood was drawn in the morning the day after admission. Peripheral blood samples were centrifuged at 3000 rpm for 5 min after standing at room temperature for at least 30 min, and the supernatant was purified. Then, the laboratory data were measured immediately. Participants were given a stool sampler and provided detailed illustrated instructions for sample collection. Stool samples freshly collected from each participant were stored with dry ice and immediately transported to the laboratory and frozen at − 80 °C immediately.

In face-to-face questionnaire interviews, we collected host metadata, including data regarding gastrointestinal distress, defecation, physical activity intensity, body weight control over one-year, dietary intake and Bristol stool score. A meal-based food frequency questionnaire (FFQ) was used to calculate baseline nutrient intake based on [China Food Composition (Book 1, 2nd Edition)]. All information provided during the interviews was recorded in the case report form.

Enzyme-linked immunosorbent assays (ELISAs) were conducted according to the manufacturer's instructions. The ELISA kits for IL-18, TNF-α, IFN-γ and high-sensitivity IL-6 were all purchased from R&D Systems, Inc. (Minneapolis, MN, USA), LPS and LBP ELISA kits were purchased from (cusabio, CSB-E09629h and CSB-E09945h). Each sample was tested three times to ensure consistency. Intra-assay precision: CV% < 8%; Inter-assay precision: CV% < 10%. We construct a standard curve by plotting the mean absorbance for each standard on

the x-axis against the concentration on the y-axis and draw a best fit curve. We make sure the levels were within the standard curve according to the manufacturer's instructions.

Follow-up and Outcomes

Follow-up was conducted by clinical visit or telephone and medical chart abstraction to determine adverse outcomes. The median follow-up time was 1.85 years (interquartile range: 1.5–2.15 years). The primary endpoint was a composite of all-cause death, nonfatal myocardial infarction, and nonfatal stroke. Prespecified main secondary endpoints were any coronary heart disease event including unstable angina, arrhythmia and congestive heart failure requiring hospitalization or an ischemia-driven coronary revascularization procedure such as invasive coronary angiography, percutaneous transluminal coronary intervention and coronary artery bypass graft. Medical records were accessed or requested to validate all self-reported events. The classification of events and follow-up data was made on the basis of phone interview, electronic medical record review. Missing rate is 13.1%, including wrong number, refusal of follow-up, transfer to other medical centers. Adjudication was conducted by 2 independent physicians who were blinded to the study.

Metagenomics analysis

DNA extraction and library construction.

Total bacterial DNA from each fecal sample was extracted from ~200 mg of stool with the Qiagen QIAamp DNA Stool Mini Kit (Qiagen) according the manufacturer’s instructions. Qubit (Invitrogen, USA) and 1% agarose gel electrophoresis were used to analyze the quality of DNA.

The detail of DNA library construction was described as follow: 1 μg genomics DNA was randomly fragmented by Covaris and the fragmented DNAs were tested by Gel-Electrophotometric then purified by kit. The fragmented DNAs were combined with End Repair Mix, incubated at 20 °C for 30 min then purified by kit. The repaired DNAs were combined with A-Tailing Mix, and incubated at 37 °C for 30 min. Illumina adaptors were ligated to the Adenylated 3'Ends DNA, and incubated at 16 °C for 16 h, then purified the DNA with kit. After selecting the correct insert size DNA fragments, several rounds of PCR amplification with PCR Primer Cocktail and PCR Master Mix were performed to enriched the Adapter-ligated DNA fragments, the purified the PCR products with kit. AxyPrep Mag PCR Clean-up Kit (Axygen) was use to purify DNA in all steps of DNA library construction. The final DNA libraries were determined the average insert size using the Agilent 2100 Bioanalyzer (Agilent Technologies, USA) and quantified by ABI StepOnePlus Real-Time PCR system (Applied Biosystems, USA).

Metagenomic sequencing and data quality control.

Samples were sequenced on the Illumina HiSeq X Ten platform with an insert size of 300 bp (paired end, 150 bases pairs). Before further bioinformatic analysis, raw reads containing adaptor sequence, low quality (lower Q score 20 rate > 50%) and ambiguous bases (N base rate less than 5%) were filtered out with SOAPnuke v1.5.6 [4]. 2.6 Tb of high-quality PE reads for the 319 samples were acquired with an average of 8.2 Gb per sample (Table S4). In order to remove human host DNA contamination, reads were aligned to human genome reference (hg19) by SOAPaligner v2.22 [5] (parameters: -m 280 -x 420 -r 1 -l 32 -s 75 -c 0.9) and the mapping reads were discarded. The average rate of host contamination was 0.05 ± 0.22%.

Metagenome assembly and binning.

We co-assembled high-quality paired-end reads from each of the 319 samples using MegaHIT v.1.1.3 [6] (options '--k-min 31 --k-max 111 --k-step 20 -t 10') into contigs. Contigs shorter than 2,000 nt were discarded from further processing. This resulted 74.41% of contigs longer than 3000bp on average, totally 49.054× 10^9 bp, with an average N50 of 8668.72 bp. The co-assembled contigs were binned with three different tools, MetaBAT2 (version 2), MaxBIN (version 2.2.6), and CONCOCT (version 1.0.0) using the default settings in the "Binning module" implemented in MetaWRAP v1.2. The metagenome-assembled genomes (MAGs) generated with the above three different algorithms were consolidated with the "Bin_refinement module" in MetaWRAP [7] using a minimum cutoff value of 50% for completeness and a maximum cutoff value of 10% for contamination (Table S5).

Completeness and contamination levels of genomic MAGs were estimated through the CheckM v1.0.7 [8]. The 319 independently assembled MAGs set were dereplicated with dRep v2.3.2 [9] using a 95% threshold (parameters: -sa 0.95 -nc 0.6 --ignoreGenomeQuality -p 6), which resulted in 1046 dereplicated MAGs. Salmon v0.14.3 [10] was used to quantify the raw read count for each MAG (parameters: --libType IU --meta -p 3). Taxonomy was performed by matching non-redundant bins with NCBI genome database available as of August 2019 (http://www.ncbi.nlm.nih.gov/, including Bacteria, Archaea, fungi and viruses).

PcoA diversity analysis

Principal coordinates analysis (PCoA) was performed based on MAG-level Bray–Curtis dissimilarity to visualize between-sample variation in gut microbiome composition. Permutational multivariate analysis of variance (PerManova/Adonis) [11] implemented in R

was used to test differences in β-diversity between predefined study subgroups, with 9,999 permutations. To evaluate host factors associated with gut microbiome β-diversity, we performed a univariable PerManova/Adonis screening analysis. Each host factor was tested separately against the Bray–Curtis dissimilarity matrix, and the resulting R^2 values represent marginal explanatory fractions from one-factor models. *p* values from the host-factor screening analysis were adjusted across the tested variables using the Benjamini–Hochberg method and reported as *q* values in Table S6.

Co-abundance group (CAG) generation of MAGs using SparCC

The correlation among 1046 MAGs was calculated by the SparCC algorithm [12] with a bootstrap procedure repeated 100 times, and then correlation matrices were computed from the resampled data matrices. Once the bootstrapped correlation scores have been computed, only bins with correlation scores greater than 0.4 were classified into bin clusters. Meanwhile, we threshold the *p* value at the desired cut-off < 0.05. The correlation values were converted to a correlation distance (1-correlation value), and the bins were clustered using the Ward clustering algorithm [13] via the R package WGCNA [14]. Various stages of CAD were compared with Con and to each other using the Wilcoxon test and T test, CAGs between the four groups were reserved when the Kruskal-Wallis test satisfied *p* value < 0.05. The bin clusters network was visualized in Cytoscape (version 3.2.1).

De novo non-redundant metagenomic gene-catalogue construction and gene-abundance-profile calculations.

Prediction of genes from each sample was processed based on Meta-GeneMark v.3.26 [15]

(parameters： -a -d -f -G) program. A non-redundant gene catalogue of 1,907,752 microbial genes was constructed with CD-HIT v4.6.4 using the parameters “-aS 0.9 -c 0.95 -M 0 -d 0 -g 1 -T 12”. The effective high-quality reads were mapped to the nonredundant unique genes using Salmon v0.14.3 to finally generate the nonredundant unique gene catalogs containing gene-abundant information. Finally, we constructed 0.396 million genes per sample on average.

Functional Annotation

KEGG databases (release 89.1) included a large of important information about the biological system, hence providing an avenue to understand the microbiome functions in hosts from metagenomic datasets. We mapped sequences of gene catalog into the KO in KEGG databases using DIAMOND [16] program with default parameters. We summed the relative abundance of genes from the same KO to yield the abundance of that KO using previously published methods [17]. The relative abundance of an enzyme was calculated from summation of the relative abundance of its corresponding KOs.

Multi-omics correlation analysis (Linkage KEGG module to phenotype of interest)

We use the previously methods to filter the KEGG module with clinical phenotype [18]. Since each KEGG module is constituted of multiple KOs. Thus, to generate results on the level of modules, we test if correlations between the phenotype and the abundances of KOs in the module are significantly higher or lower (Mann-Whitney U test) for the module member KOs than for all other KOs, thus also considering module completeness beyond the single-gene level. Correlation analysis of the gut microbiome and host metabolome were investigated by nonparametric Spearman’s correlation analysis. Microbial and functional profiles were

analyzed as relative abundance profiles after sample-wise normalization. Features with excessive sparsity were excluded before downstream association analyses. Rank-based non-parametric tests or Spearman correlation analyses were used where appropriate.

CAG contribute to KEGG module

To identify the bin cluster that contributed the most to the association between a given functional KEGG module and CAD phenotype, we performed analysis based on a variation of the leave-one-out principle [18], effectively evaluating the importance of each bin cluster by the difference in association strength that results from removing it from the analysis. It is done by testing for each KEGG module to what extent leaving out each bin cluster and the genes it contains causes a change in the association between those modules and the target clinical phenotype.

Random survival forest analysis

We tested the relation of the bacterial markers with cardiac outcomes using multivariate random survival forest [19] (R version 3.6.3 package randomForestSRC). Based on the abundance profiles of CAGs and KEGG modules, we constructed RSF models using the randomForestSRC package with the log-rank splitting rule. Predictor importance was estimated by repeated RSF modeling using ensemble permutation importance. Candidate predictor sets were generated by sequentially adding predictors according to their median importance. For each candidate set, model performance was assessed using OOB-predicted risk scores and Harrell's C-index across repeated RSF iterations. The predictor set with the highest median OOB C-index was selected for the final model. Average OOB mortality scores from repeated

RSF runs were used to stratify patients into risk groups. The cut-off for dichotomization was determined using survival-based cutpoint analysis, and the association between the resulting risk groups and MACE was evaluated using Kaplan–Meier analysis and multivariable Cox proportional hazards regression. We sequentially added the survival predictors in decreasing order of median ensemble permutation importance. To obtain stable performance estimates of the RSF classifier, we then computed median out-of-bag (OOB) concordance index at each step of variable inclusion of RSF modeling. The top survival predictors that maximized the performance of the RSF model were selected for inclusion in the final model. We selected an optimal cut-value for dichotomization of average OOB mortality scores by maximization of hazard ratio significance through iterative log-rank tests. Finally, we confirmed the prognostic significance of stratified risk groups through analyses of Kaplan-Meier curve and multivariate Cox proportional hazards regression.

Metabolomics analysis

Analysis of fasting serum non-targeted metabolites by UHPLC × QTOFMS.

Sample analysis was performed on Waters ACQUITY ultra-high-performance liquid chromatography system (Milford, MA) coupled with a Waters Q-TOF Micromass system (Manchester, UK). Detailed parameters for the sample preparation and HPLC-MS experiment parameters were detailed described in our previous research [20]. The raw data were imported to the Progenesis QI (Waters) for peak alignment to obtain a peak list containing the retention time, m/z, and peak area of each sample [21]. By using retention time and the m/z data pairs as the identifiers for each ion, we obtained ion intensities of each peak and generated a matrix containing arbitrarily assigned peak indices (retention time-m/z pairs), ion intensities (variables)

and sample names (observations). The matrix was further reduced by removing peaks with missing values in more than 80% samples and those with isotope ions from each group to obtain consistent variables. The CV (coefficient of variation) of metabolites in the QC samples was set at a threshold of 30%, as a standard in the assessment of repeatability in metabolomics data sets. The nonparametric univariate method (Mann-Whitney-Wilcoxon test) was used to analyze metabolites that differed in abundance between the different subgroups corrected for false discovery rate (FDR) to ensure that the peak of each metabolite was reproducibly detected in the samples. The online HMDB database (version: 4.0) [22] and KEGG database (updated: September 14, 2016) [23], Lipid maps Structure Database (LMSD, updated: October, 2017) [24] and METLIN (version: 1.0.5673.40082) [25] were used to align the molecular mass data (m/z) to identify metabolites.

Clustering of co-abundant serum metabolites.

A total of 7061 serum metabolic features were yielded after pre-processing above. We conducted a “cross-comparison scheme” as follows to downsize metabolic features associated with disease status. *i*, Multi-comparisons set A: various stages of CAD were compared with Controls and to each other using Wilcoxon test and T test, the metabolites will be retained for the next stage of analysis both statistics results satisfied the adjusted p value < 0.05 meanwhile the fold change > 1.2 or < 0.83; *ii*, Multi-comparisons set B: compare the metabolites between the four groups and Con vs. CAD, metabolites were reserved when any test of Jonckheere-Tespstra test or Kruskal.test satisfied p value < 0.05. We then integrated the two collections, serum metabolomics profiling yielded 562 features after removing the drug metabolites. Clusters of co-abundant serum metabolites were identified using the R package WGCNA [14].

Signed, weighted metabolite co-abundance correlation networks were calculated for all examined individuals. A scale-free topology criterion was used to choose the soft threshold $\beta = 7$ for serum metabolites correlations. Clusters were identified with the dynamic hybrid tree-cutting algorithm using a deepSplit of 4 [13]. The serum metabolites clusters (labelled M01–M35) were collectively termed serum metabotypes.

Analysis of targeted aromatic amino acid panel metabolites for both human and mice

Mouse stool was weighed and mixed with extraction solvent of ice-cold 50% acetonitrile (v/v) containing 1 μM chloropropamide (internal standard) in 1/2 ratio (Ws/Vs). The mixture was homogenized with 1 mm zirconium beads using a BeadBlaster^{TM} homogenizer followed by sonication for 20 min in an ice bath. 20 μL human serum were mixed with 80 uL ice-cold 50% acetonitrile (v/v) containing 1 μM chloropropamide. All samples were homogenized according to the program parameters: 6500–1 × 30–005 (×3). Following centrifugation at 20,000 × g at 4°C for 20 min, the supernatants were collected and diluted by 5-fold and 50-fold with pure water containing 1 μM chlorpropamide before being subjected to analysis. Targeted quantitation was performed to determine the concentration of AAAs pathway metabolites as previously described [26] by reverse- phase UHPLC using a Prominence 20 UFLCXR system (Shimadzu, Columbia, MD, USA) with a Waters (Milford, MA, USA) BEH C18 column (2.1 × 100 mm × 1.7 μm particle size) maintained at 55°C and a 20 min aqueous acetonitrile gradient, at a flow rate of 250 μL/min.

Animal experiment

Mice and diets

Both specific pathogen-free (SPF) and germ-free (GF) *ApoE* $^{-/-}$ mice were generated and provided by the Institute of Laboratory Animal Sciences (ILAS) at the Chinese Academy of Medical Sciences and Peking Union Medical College [research license no. SYXK (Beijing) 2015-0035], which is a member of (and accredited by) the American Association for the Accreditation of Laboratory Animal Care. All experiments were performed in accordance with the guidelines of the Institutional Animal Care and Use Committees of the ILAS. The mice were maintained under standard specific pathogen-free (conventionally raised) or germ-free (GF) conditions with a 12:12-hour light: dark cycle. GF mice were maintained in flexible film isolators and their GF status was checked by aerobic and anaerobic culture. Surveillance for bacterial contamination was performed by periodic bacteriological examinations of feces, food and padding. Normal saline was added into the samples with sufficient mixing. The mixtures were then cultured using the spread plate method on: 1) Brain Heart Infusion agar and Thioglycolate agar under aerobic condition at 37°C for aerobic bacteria; 2) on sheep blood agar plates and incubate at 37°C for 48 hours; 3) on Trypticase soy broth under aerobic condition at 25-28°C for fungi. All cultures were examined under optical microscope after 1, 4, 7 and 14 days. All SPF *ApoE* $^{-/-}$ mice were male while GF *ApoE* $^{-/-}$ mice were gender matched. All mice were used after reaching the age of 6 weeks. Both SPF and GF *ApoE* $^{-/-}$ mice received sterilized high fat diet (40 kcal% fat and 1.25% w/w cholesterol; product number D12108S) which was purchased from Changzhou SYSE Bio-Tec. Co., Ltd.

Intestinal microbiota depletion with Abx treatment

SPF *ApoE* $^{-/-}$ mice were treated by broad-spectrum antibiotics for 4 weeks containing vancomycin 500mg/L, metronidazole 1g/L, neomycin 1g/L and penicillin 1g/L, which were added in the sterile drinking water. Solutions and bottles were changed three times per week. All antibiotics were purchased from Macklin (Shanghai, China). Abx activity was confirmed by analyzing faeces for the presence of bacterial DNA by qPCR. qPCR was performed by StepOnePlusTM Real-Time PCR System (Applied Biosystems, Foster City, CA, USA) with TB Green Premix Ex TaqTM (Takara Bio, Inc.) the following parameters: a single cycle at 95°C for 30 s, then 35 cycles at 95°C for 5 s, and 60°C for 30 s. The total bacterial number in feces was analyzed by qPCR using the following universal primers: forward, 50 -TCCTACGGGAGGCAGCAGT-30, reverse, 50-GGACTACCAGGGTATCTAATCCTGTT-30.

Fecal microbiota transplantation

We colonized Abx treated SPF and GF *ApoE* $^{-/-}$ mice with fecal microbiota from Control donors (Con, N = 5) and coronary artery patients belong to 3-VD group (3-VD, N = 5) from the current cohort. The appropriate number of donors was determined on the basis of previous FMT experiments [27]. We conducted donors screening according to the following standards: *i*. We selected 3-VD patients who were also diagnosed with myocardial infarction, since their clinical symptoms were more severe and typical; *ii*. Donors were selected to be representative of the 3-VD cohort microbiota signature, based on 16S rRNA sequencing profiles. Adult Abx-treated SPF *ApoE* $^{-/-}$ mice were divided into 2 groups and received long term (20 weeks) microbiota colonization: (*i*) Con group, gavaged with faeces mixture from control donors; and (*ii*) 3-VD group, gavaged with faeces mixture from 3-VD patients. Adult GF *ApoE* $^{-/-}$ mice were also

divided into 2 groups as Con group (n = 8, male; n = 9, female) and 3-VD group (n = 6, male; n = 6, female) receiving same faeces mixture gavage for 4 weeks.

Each fecal sample (0.5 g) was diluted in 25 mL of a sterile Ringer working buffer (9 g/L of sodium chloride, 0.4 g/L of potassium chloride, 0.25 g/L of calcium chloride dehydrate and 0.05% (w/v) L-cysteine hydrochloride) in an anaerobic chamber (80% N_2: 10% CO_2: 10% H_2). The fecal material was suspended by thorough vortexing (5 mins) and settled by gravity for 5 min. The clarified supernatant was transferred to a clean tube, and an equal volume of 20% (w/v) skimmed milk (232100, BD) was added. The inoculum was freshly prepared on the day of experiment, with the rest stored at -80°C until the second inoculation. 200 μL of fecal water was administered in a single dose by oral gavage per mouse. To ensure the continuous effect of the microbiota transplantation, after 5 oral gavages, once every two days (days 1, 3, 5, 7, and 9), the mice were then orally gavaged once a week until the end of the experiment (SPF mice, 20 weeks; GF mice, 4 weeks). Finally, mice were anesthetized by i.p. injection of chloral hydrate (300 mg/kg).

16S rRNA sequencing for host donors and receipt mice

Total genome DNA from samples was extracted using CTAB/SDS method. DNA concentration and purity was monitored on 1% agarose gels. According to the concentration, DNA was diluted to 1ng/μL using sterile water. 16S rRNA genes of V3-V4 were amplified used specific primer with the barcode. All PCR reactions were carried out with Phusion® High-Fidelity PCR Master Mix (New England Biolabs). Mix same volume of 1× loading buffer (contained SYB green) with PCR products and operate electrophoresis on 2% agarose gel for detection. Samples with bright main strip between 400–450bp were chosen for further experiments. PCR products was

mixed in equidensity ratios. Then, mixture PCR products was purified with Qiagen Gel Extraction Kit (Qiagen, Germany). Sequencing libraries were generated using TruSeq® DNA PCR-Free Sample Preparation Kit (Illumina, USA) following manufacturer's recommendations and index codes were added. The library quality was assessed on the Qubit@ 2.0 Fluorometer (Thermo Scientific) and Agilent Bioanalyzer 2100 system. At last, the library was sequenced on an Illumina HiSeq 2500 platform and 250 bp paired-end reads were generated.

Paired-end reads were assigned to samples based on their unique barcodes and truncated by removing the barcode and primer sequence. Microbiome bioinformatics were conducted using QIIME 2 2021.11.0 [28]. Raw sequence data were demultiplexed and adapters were removed using the 'qiime cutadapt trim-paired' command. The 'qiime dada2 denoise-paired' [29] command was used to denoise and dereplicate the paired-end sequences, which also included quality filtering and chimera filtering. All amplicon sequence variants (ASVs) were taxonomically classified using the 'qiime feature-classifier classify-sklearn' method, based on the silva_132_99_16S_341F_806R classifier trained in-house on the SILVA (V132) database [30]. Principal Coordinate Analysis (PCoA) was performed to obtain principal coordinates and visualize complex, multidimensional data. The PCoA analysis was visualized using the 'vegan' package in R (v4.3.2).

Histological study

Aortic atherosclerotic lesion area

After the mice were anesthetized, the aorta was perfused with saline and dissected from the middle of the left ventricle to the bifurcation of the iliac artery. To analyze lesions of the aortic root, samples were obtained from the portion of the ascending aorta proximal to the aortic sinus.

The samples were embedded in optimal cutting temperature compound (#4583, Sakura). Five consecutive sections (8 μm in thickness) were collected from each mouse and stained with oil red O (#G2161, Solarbio) or hematoxylin (#G1150, Solarbio). The collagen content in the plaque was assessed by Masson's trichrome staining. Immunohistochemistry was used to characterize TLR4 (dilution 1:80, 19811-1-AP, Proteintech); CD14 (dilution 1:100, 17000-1-AP, Proteintech) in atherosclerotic plaque as previously described [31]. A positive tissue control was used to ensure the specificity of antibodies used in this study. For en face lesion analysis, an aortic segment spanning from the proximal ascending aorta to the common iliac artery bifurcation was excised and fixed in 10% neutral buffered formalin. After the adventitial tissue was carefully removed, the aorta was opened longitudinally, stained with oil red O.

Quantification measurements

For cross sectional aorta and colon tissue, the stained sections were digitally scanned using Pannoramic DESK (P250, 3D HISTECH) and viewed by Caseviewer version 2.3. The en face oil red-O staining of aorta was captured using a digital camera. All measurements were performed using the ImageJ® software. For the quantitative analysis of atherosclerosis extent, the stained lesion area was reported as a percentage of the total area [32]. Collagen content of the total plaque lesion was calculated similar to the methods as previously described [33]. The IHC was expressed as the percentage of stained area to total plaque area, while quantification of the positive area in the colon was performed (10 × magnification) for the 5 separate sections obtained from each mouse.

Mouse biochemical parameters and inflammatory factors examination

All experimental mice were fasted, and blood samples were collected from the orbital vascular

plexus of mice before they were sacrificed. The whole blood samples were allowed to clot at room temperature for 1–2 hours until the serum precipitate. The supernatant was collected after centrifugation at 2000 × g for 25 min at 4°C. Several serum parameters including triglycerides, cholesterol and glucose were measured by using an automated clinical chemistry analyzer, Fuji Dri-chem N × 500 (Fujifilm Co.) according to the manufacturer's instructions. Before each measurement, a QC card (Fujifilm Co.) was used to adjust the lot variability in each of the slide reagents.

The fecal LPS levels were determined using a mouse LPS ELISA Kit (Cusabio-CSB-E13066m) according to the manufacturer's instructions. The fecal supernatant was diluted 1000–fold in pyrogen free water and inactivated for 15 minutes at 90°C. The serum samples were diluted 4–fold, then Lipopolysaccharide Binding Protein (LBP) levels were examined by ELISA kit (CKM043, cellsciences). Serum multiple inflammatory factors were measured by Meso Scale Discovery (MSD)'s immunoassay kit (K15048D-1) equipped with Meso QuickPlex SQ 120 following the manufacturer's instructions. All measurements were performed in pyrogenfree glass tubes, Eppendorf tubes, and plates.

Isolation of total RNA and quantitative reverse transcription PCR (RT-qPCR) analysis

Total RNA was extracted from the aorta using an RNeasy® Mini kit following the manufacturer's instructions. cDNA synthesis was performed using PrimeScriptTM RT Master Mix (TaKaRa Bio., Japan). Then, qRT–PCR was performed using an Applied Biosystem StepOne Plus PCR system with TB GreenTM Premix Ex TaqTM II (TaKaRa Bio., Japan). Gene expression levels were determined using the comparative ΔΔCT method.

Transcriptome study

The concentration and quality of the purified RNA was determined using a NanoDrop ND-1000 instrument (Thermo Scientific, United States) and an Agilent Bioanalyzer 2100 system (Agilent Technologies, United States), respectively. The sequencing libraries were generated according to the Illumina TrueSeq protocol. Paired-end RNA sequencing was performed on an Illummina HiSeq 2500 instrument following the manufacturer's instructions with a quality control standard (for each sample, the sequencing depth was higher than 6 GB, the read length was higher than 90 nt, and the Q30 > 85%). Raw data (raw reads) in the fastq format were first processed using in-house Perl scripts. Clean data (clean reads) were then obtained by removing reads containing adapter sequences and poly-N sequences from the raw data.

The high-quality reads from each sample were aligned to the Mus musculus reference genome, GRCm38 (Genome Reference Consortium mouse build 382), using hierarchical indexing for spliced alignment of transcripts (HISAT2) with the default parameters [34]. The unambiguous alignments from each sample were quantified using featureCounts [35], and the gene expression profile with respect to the read counts was acquired. Differentially expressed genes in the two groups were determined by using the DESeq2 package [36] in R. Gene ontology (GO) enrichment and (Kyoto encyclopedia of genes and genomes (KEGG) pathway analysis of differentially expressed genes was performed using the clusterProfiler package [37]. Significant differences are presented as the adjusted p value.

Immune cell isolation

Immune cell isolation from peripheral blood, aorta, and spleen was performed in the current study. For GF *ApoE* $^{-/-}$ mice, blood (800–1000 μL) was collected into EDTA containing tubes,

centrifuge at 3000 rpm for 10 min at 4°C, aspirate the upper layer of plasma for later use. Blood cells were resuspended with RBC lysis buffer (7 mL, BD Bio-sciences, USA), rotated for 5 minutes at 4°C. Centrifugation (4°C, 300g, 5min) and discard the supernatant, repeat this step until the red blood cells were cleavage completely. Then resuspend the cell pellet in 1mL 5%FBS/PBS buffer.

The methodology to isolate and characterize the immune cells within the aorta were same as previously with some modifications [38]. Briefly, the aorta was carefully dissected and cut into small pieces. Then the tissues underwent enzymatic digestion including 450 U/ml collagenase type I, 250 U/ml collagenase type XI, 120 U/ml hyaluronidase type I-s and 120 U/ml DNAse1 and incubate for 30–45 minutes at 37°C. Add 2 volumes of 5% FBS-RPMI to stop the enzymatic hydrolysis reaction. Use 40 μm cell strainer and syringe to release cells from digested aortic tissue returning cells to FACS tube.

Spleens were removed and cut into pieces in cold PBS. After filtration through a 200-gauge steel mesh and the removal of red blood cells with RBC lysis buffer, splenocytes were collected for further experimentation.

Flow cytometry

All peripheral blood cells, aortic cells and splenocytes were treated with Zombie Aqua fixable viability dye to determine live/dead cells, and were incubated with CD16/CD32 mAb (clone 2.4G2, BioLegend, USA) for 30 min to block Fc receptors. Then different kind of cell pellets were subsequently stained with different antibody cocktails for analysis by flow cytometry. Flow cytometry was conducted with LSRFortessa SORP using BD FACSDiva software (15013129; BD Biosciences). For blood and aortic cells, cell populations were discriminated

by the following antibody cocktail: anti-CD45 (103116, Biolegend; 1:100, 1µl per test), anti-CD4 (100443, Biolegend; 1:50, 2µl per test), anti-CD8 (100734, Biolegend; 1:33, 3µl per test), anti-CD11b (101212, Biolegend; 1:100, 1µl per test), anti-Ly-6G (127643, Biolegend; 1:50, 2µl per test), anti-F4/80 (123110, Biolegend; 1:33, 3µl per test), anti-CD11c (117318, Biolegend; 1:100, 1µl per test) and anti-Ly-6C (128006, Biolegend; 1:200, 0.5µl per test).

For splenocytes, cell pellets were resuspended in staining buffer and incubated with a cocktail of anti-mouse CD45 (103116, Biolegend; 1:100, 1µl per test), CD4 (100443, Biolegend; 1:50, 2µl per test), CD8 (100734, Biolegend; 1:33, 3µl per test), IFN-γ (505826, Biolegend; 1:20, 5µl per test), IL-17a (506930, Biolegend; 1:100, 1µl per test), IL-4 (504133, Biolegend; 1:50, 2µl per test), CD25 (101904, Biolegend; 1:20, 5µl per test) and Foxp3 (126408, Biolegend; 1:50, 2µl per test) fluorochrome-conjugated antibodies. To perform intracellular staining, cells were treated using a Fixation/Permeabilization Solution kit (BD Pharmingen™, USA) and then stained with the appropriate antibodies. The detailed procedures were summarized as previously described [39]. The gating strategy were listed in Figure S11. Cell populations and marker expression were gated using the FlowJo analysis program (ver. 10.4).

Statistical analysis

GraphPad Prism version 9.0 (GraphPad Software) and SPSS version 25.0 were used for statistical analysis. The sample distribution was determined by the Kolmogorov–Smirnov normality test. Continuous variables were expressed as means with standard deviations (means ± s.e.m.) in the case of normal distribution or medians with interquartile ranges when variables were not normally distributed. One-way analysis of variance (ANOVA) followed by Tukey's post-hoc test was used to evaluate the statistical significance of differences among three or more

groups. The Kruskal–Wallis test was used to analyze differences among three or more experimental groups, followed by Dunn's postdoc analysis. Two-tailed Student's t-test was used to evaluate statistical significance between two groups. For the nonparametric tests, the two-tailed Mann–Whitney U-test was used to evaluate statistical significance between two groups. Categorical data are expressed as numbers and percentages and compared with the chi-squared test or Fisher's exact test. Two-tailed paired-samples t-tests were applied to analyze the paired groups. Two-tailed Wilcoxon matched-pairs signed rank tests were applied to analyze the paired groups. For most analyses of outcomes, we divided metabolites or bacteria levels into quartiles (high vs. low). We used Kaplan–Meier analysis with Cox proportional- hazards regression for the time-to-event analysis to determine hazard ratios and 95% confidence intervals for major adverse cardiovascular events. We applied methods unaffected by the missing values.

Multiple-testing correction was performed within each predefined analysis family using the Benjamini–Hochberg method. These analyses included host-factor PerManova/Adonis screening, CAG differential-abundance analyses, CAG–phenotype correlations, KEGG module-level associations, untargeted metabolomic feature screening, and serum metabotype–phenotype correlations. Unless otherwise specified, FDR-adjusted $q < 0.05$ was considered statistically significant, while $q < 0.1$ was considered suggestive in exploratory correlation heatmaps.

References

1. Neeland, Ian J., Riyaz S. Patel, Parham Eshtehardi, Saurabh Dhawan, Michael C. McDaniel, S. Tanveer Rab, Viola Vaccarino, et al. 2012. "Coronary angiographic scoring systems: An evaluation of their equivalence and validity." *American Heart Journal* 164: 547-552. https://doi.org/10.1016/j.ahj.2012.07.007

2. Gensini, G. G. 1983. “A more meaningful scoring system for determining the severity of coronary heart disease.” *American Journal of Cardiology* 51: 606.

3. Sianos, Georgios, Marie-Angèle Morel, Arie Pieter Kappetein, Marie-Claude Morice, Antonio Colombo, Keith Dawkins, Marcel van den Brand, et al. 2005. “The SYNTAX Score: an angiographic tool grading the complexity of coronary artery disease.” *EuroIntervention* 1: 219-227.

4. Chen, Yuxin, Yongsheng Chen, Chunmei Shi, Zhibo Huang, Yong Zhang, Shengkang Li, Yan Li, et al. 2018. “SOAPnuke: a MapReduce acceleration-supported software for integrated quality control and preprocessing of high-throughput sequencing data.” *Gigascience* 7: 1-6. https://doi.org/10.1093/gigascience/gix120

5. Li, Ruiqiang, Yingrui Li, Karsten Kristiansen, Jun Wang. 2008. “SOAP: short oligonucleotide alignment program.” *Bioinformatics* 24: 713-714. https://doi.org/10.1093/bioinformatics/btn025

6. Li, Dinghua, Chi-Man Liu, Ruibang Luo, Kunihiko Sadakane, Tak-Wah Lam. 2015. “MEGAHIT: an ultra-fast single-node solution for large and complex metagenomics assembly via succinct de Bruijn graph.” *Bioinformatics* 31: 1674-1676. https://doi.org/10.1093/bioinformatics/btv033

7. Uritskiy, Gherman V, Jocelyne DiRuggiero, James Taylor. 2018. “MetaWRAP-a flexible pipeline for genome-resolved metagenomic data analysis.” *Microbiome* 6: 158. https://doi.org/10.1186/s40168-018-0541-1

8. Parks, Donovan H, Michael Imelfort, Connor T Skennerton, Philip Hugenholtz, Gene W Tyson. 2015. “CheckM: assessing the quality of microbial genomes recovered from isolates, single cells, and metagenomes.” *Genome Research* 25: 1043-1055. https://doi.org/10.1101/gr.186072.114

9. Olm, Matthew R, Christopher T Brown, Brandon Brooks, Jillian F Banfield. 2017. “dRep: a tool for fast and accurate genomic comparisons that enables improved genome recovery from metagenomes through de-replication.” *The ISME Journal* 11: 2864-2868. https://doi.org/10.1038/ismej.2017.126

10. Patro, Rob, Geet Duggal, Michael I Love, Rafael A Irizarry, Carl Kingsford. 2017. “Salmon provides fast and bias-aware quantification of transcript expression.” *Nature Methods* 14: 417-419. https://doi.org/10.1038/nmeth.4197

11. Anderson, Marti J. 2017. Permutational Multivariate Analysis of Variance (PERMANOVA). *Wiley StatsRef: Statistics Reference Online* 1-15. https://doi.org/10.1002/9781118445112.stat07841

12. Friedman, Jonathan, Eric J Alm. 2012. “Inferring correlation networks from genomic survey data.” *PLOS Computational Biology* 8: e1002687. https://doi.org/10.1371/journal.pcbi.1002687

13. Langfelder, Peter, Bin Zhang, Steve Horvath. 2008. “Defining clusters from a hierarchical cluster tree: the Dynamic Tree Cut package for R.” *Bioinformatics* 24: 719-720. https://doi.org/10.1093/bioinformatics/btm563

14. Langfelder, Peter, Steve Horvath. 2008. “WGCNA: an R package for weighted correlation network analysis.” *BMC Bioinformatics* 9: 559. https://doi.org/10.1186/1471-2105-9-559

15. Zhu, Wenhan, Alexandre Lomsadze, Mark Borodovsky. 2010. “Ab initio gene

identification in metagenomic sequences." *Nucleic Acids Research* 38: e132-e132. https://doi.org/10.1093/nar/gkq275

16. Buchfink, Benjamin, Chao Xie, Daniel H Huson. 2015. "Fast and sensitive protein alignment using DIAMOND." *Nature Methods* 12: 59-60. https://doi.org/10.1038/nmeth.3176

17. Li, Junhua, Huijue Jia, Xianghang Cai, Huanzi Zhong, Qiang Feng, Shinichi Sunagawa, Manimozhiyan Arumugam, et al. 2014. "An integrated catalog of reference genes in the human gut microbiome." *Nature Biotechnol* 32: 834-841. https://doi.org/10.1038/nbt.2942

18. Pedersen, Helle Krogh, Sofia K Forslund, Valborg Gudmundsdottir, Anders Ostergaard Petersen, Falk Hildebrand, Tuulia Hyotylainen, Trine Nielsen, et al. 2018. "A computational framework to integrate high-throughput '-omics' datasets for the identification of potential mechanistic links." *Nature Protocols* 13: 2781-2800. https://doi.org/10.1038/s41596-018-0064-z

19. Hemant, Ishwaran, B. Kogalur Udaya, H. Blackstone Eugene, S. Lauer Michael. 2008. "Random survival forests." *The Annals of Applied Statistics* 2: 841-860. https://doi.org/10.1214/08-AOAS169

20. Liu, Honghong, Xi Chen, Xiaomin Hu, Haitao Niu, Ran Tian, Hui Wang, Haiyu Pang, et al. 2019. "Alterations in the gut microbiome and metabolism with coronary artery disease severity." *Microbiome* 7: 68. https://doi.org/10.1186/s40168-019-0683-9

21. Li, Yanjie, Xue Song, Xinjie Zhao, Lijuan Zou, Guowang Xu. 2014. "Serum metabolic profiling study of lung cancer using ultra high performance liquid chromatography/quadrupole time-of-flight mass spectrometry." *Journal of Chromatography B: Analytical Technologies in the Biomedical and Life Sciences* 966: 147-153. https://doi.org/10.1016/j.jchromb.2014.04.047

22. Wishart, David S, Yannick Djoumbou Feunang, Ana Marcu, An Chi Guo, Kevin Liang, Rosa Vazquez-Fresno, Tanvir Sajed, et al. 2018. "HMDB 4.0: the human metabolome database for 2018." *Nucleic Acids Research* 46: D608-d617. https://doi.org/10.1093/nar/gkx1089

23. Kanehisa, Minoru, Susumu Goto. 2000. "KEGG: Kyoto Encyclopedia of Genes and Genomes." *Nucleic Acids Research* 28: 27-30. https://doi.org/10.1093/nar/28.1.27

24. Sud, Manish, Eoin Fahy, Dawn Cotter, Alex Brown, Edward A Dennis, Christopher K Glass, Alfred H Jr Merrill, et al. 2007. "LMSD: LIPID MAPS structure database." *Nucleic Acids Res* 35: D527-532. https://doi.org/10.1093/nar/gkl838

25. Smith, Colin A, Grace O'Maille, Elizabeth J Want, Chuan Qin, Sunia A Trauger, Theodore R Brandon, Darlene E Custodio, Ruben Abagyan, Gary Siuzdak. 2005. "METLIN: a metabolite mass spectral database." *Therapeutic Drug Monitoring* 27: 747-751. https://doi.org/10.1097/01.ftd.0000179845.53213.39

26. Dong, Fangcong, Fuhua Hao, Iain A. Murray, Philip B. Smith, Imhoi Koo, Alyssa M. Tindall, Penny M. Kris-Etherton, et al. 2020. "Intestinal microbiota-derived tryptophan metabolites are predictive of Ah receptor activity." *Gut Microbes* 12: 1788899. https://doi.org/10.1080/19490976.2020.1788899

27. De Palma, Giada, Michael D. J. Lynch, Jun Lu, Vi T. Dang, Yikang Deng, Jennifer

Jury, Genevieve Umeh, et al. 2017. "Transplantation of fecal microbiota from patients with irritable bowel syndrome alters gut function and behavior in recipient mice." *Science Translational Medicine* 9: eaaf6397. https://doi.org/10.1126/scitranslmed.aaf6397

28. Bolyen, Evan, Jai Ram Rideout, Matthew R. Dillon, Nicholas A. Bokulich, Christian C. Abnet, Gabriel A. Al-Ghalith, Harriet Alexander, et al. 2019. "Reproducible, interactive, scalable and extensible microbiome data science using QIIME 2." *Nature Biotechnology* 37: 852-857. https://doi.org/10.1038/s41587-019-0209-9

29. Callahan, Benjamin J., Paul J. McMurdie, Michael J. Rosen, Andrew W. Han, Amy Jo A. Johnson, Susan P. Holmes. 2016. "DADA2: High-resolution sample inference from Illumina amplicon data." *Nature Methods* 13: 581-583. https://doi.org/10.1038/nmeth.3869

30. Quast, Christian, Elmar Pruesse, Pelin Yilmaz, Jan Gerken, Timmy Schweer, Pablo Yarza, Jörg Peplies, Frank Oliver Glöckner. 2013. "The SILVA ribosomal RNA gene database project: improved data processing and web-based tools." *Nucleic Acids Research* 41: D590-D596. https://doi.org/10.1093/nar/gks1219

31. Huang, Zhouqing, Liansheng Wang, Shu Meng, Yue Wang, Ting Chen, Changqian Wang. 2011. "Berberine reduces both MMP-9 and EMMPRIN expression through prevention of p38 pathway activation in PMA-induced macrophages." *International Journal of Cardiology* 146: 153-158. https://doi.org/10.1016/j.ijcard.2009.06.023

32. Au - Centa, Monica, Daniel F. J. Au - Ketelhuth, Stephen Au - Malin, Anton Au - Gisterå. 2019. "Quantification of Atherosclerosis in Mice." *JoVE* e59828. https://doi.org/doi:10.3791/59828

33. Chen, Ying, Qi Yu, Cang-Bao Xu. 2017. "A convenient method for quantifying collagen fibers in atherosclerotic lesions by ImageJ software." *International Journal of Clinical and Experimental Medicine* 10: 14927-14935.

34. Kim, Daehwan, Ben Langmead, Steven L Salzberg. 2015. "HISAT: a fast spliced aligner with low memory requirements." *Nature Methods* 12: 357-360. https://doi.org/10.1038/nmeth.3317

35. Liao, Yang, Gordon K Smyth, Wei Shi. 2014. "featureCounts: an efficient general purpose program for assigning sequence reads to genomic features." *Bioinformatics* 30: 923-930. https://doi.org/10.1093/bioinformatics/btt656

36. Love, Michael I, Wolfgang Huber, Simon Anders. 2014. "Moderated estimation of fold change and dispersion for RNA-seq data with DESeq2." *Genome Biology* 15: 550. https://doi.org/10.1186/s13059-014-0550-8

37. Yu, Guangchuang, Li-Gen Wang, Yanyan Han, Qing-Yu He. 2012. "clusterProfiler: an R package for comparing biological themes among gene clusters." *Omics* 16: 284-287. https://doi.org/10.1089/omi.2011.0118

38. Gjurich, Breanne N., Parésa L. Taghavie-Moghadam, Elena V. Galkina. 2015. Flow Cytometric Analysis of Immune Cells Within Murine Aorta. *Methods in Mouse Atherosclerosis* Springer New York, 161-175. https://doi.org/10.1007/978-1-4939-2929-0_11

39. Ma, Yiyangzi, Xiaoxue Xu, Mengtao Li, Jun Cai, Qiang Wei, Haitao Niu. 2019.

“Gut microbiota promote the inflammatory response in the pathogenesis of systemic lupus erythematosus.” *Molecular Medicine* 25: 35. https://doi.org/10.1186/s10020-019-0102-5
